# Resting-State Functional Connectivity and Network Analysis of Cerebellum with Respect to IQ and Gender

**DOI:** 10.3389/fnhum.2017.00189

**Published:** 2017-04-26

**Authors:** Vasileios C. Pezoulas, Michalis Zervakis, Sifis Michelogiannis, Manousos A. Klados

**Affiliations:** ^1^School of Electrical and Computer Engineering, Technical University of Crete Chania, Greece; ^2^Neurophysiological Research Laboratory (L. Widén), School of Medicine, University of Crete Heraklion, Greece; ^3^Max Planck Research Group for Neuroanatomy and Connectivity, Max Planck Institute for Human Cognitive and Brain Sciences Leipzig, Germany

**Keywords:** cerebellum, fMRI, small-world network, minimum spanning tree, IQ, median response time

## Abstract

During the last years, it has been established that the prefrontal and posterior parietal brain lobes, which are mostly related to intelligence, have many connections to cerebellum. However, there is a limited research investigating cerebellum's relationship with cognitive processes. In this study, the network of cerebellum was analyzed in order to investigate its overall organization in individuals with low and high fluid Intelligence Quotient (IQ). Functional magnetic resonance imaging (fMRI) data were selected from 136 subjects in resting-state from the Human Connectome Project (HCP) database and were further separated into two IQ groups composed of 69 low-IQ and 67 high-IQ subjects. Cerebellum was parcellated into 28 lobules/ROIs (per subject) using a standard cerebellum anatomical atlas. Thereafter, correlation matrices were constructed by computing Pearson's correlation coefficients between the average BOLD time-series for each pair of ROIs inside the cerebellum. By computing conventional graph metrics, small-world network properties were verified using the weighted clustering coefficient and the characteristic path length for estimating the trade-off between segregation and integration. In addition, a connectivity metric was computed for extracting the average cost per network. The concept of the Minimum Spanning Tree (MST) was adopted and implemented in order to avoid methodological biases in graph comparisons and retain only the strongest connections per network. Subsequently, six global and three local metrics were calculated in order to retrieve useful features concerning the characteristics of each MST. Moreover, the local metrics of degree and betweenness centrality were used to detect hubs, i.e., nodes with high importance. The computed set of metrics gave rise to extensive statistical analysis in order to examine differences between low and high-IQ groups, as well as between all possible gender-based group combinations. Our results reveal that both male and female networks have small-world properties with differences in females (especially in higher IQ females) indicative of higher neural efficiency in cerebellum. There is a trend toward the same direction in men, but without significant differences. Finally, three lobules showed maximum correlation with the median response time in low-IQ individuals, implying that there is an increased effort dedicated locally by this population in cognitive tasks.

## Introduction

During the last decades, many neuroimaging studies have been performed toward establishing the relationship between brain volume, connectivity structures and intelligence. It is obvious now that the human intelligence, which is a general cognitive mental ability, depends on structural and functional properties of the brain, as well as on the interaction among different brain regions (Jung et al., 1999; Duncan et al., 2000; Shaw et al., 2006). Findings support the importance of prefrontal cortex and regions of parietal lobes for intelligence (Duncan, 1995; Jung and Haier, 2007; Song et al., 2008; Deary et al., 2010; Ryman et al., 2016). Gray and white-matter characteristics have been used to study the correlation between structural findings and intellectual abilities (Mechelli et al., 2005; Hulshoff Pol et al., 2006; Choi et al., 2008; Malpas et al., 2016), while studies associating anatomical and functional connectivity with intelligence have been also reported (Haier et al., 2005; Song et al., 2008; Chiang et al., 2009; Ryman et al., 2016; Tsvetanov et al., 2016), with indicative biomarkers involving the total brain volume and the concentration of the N-acetyl aspartate (McDaniel, 2005; Paul et al., 2016).

The organized network activity at rest could be viewed as the idle state of the brain functions engaged during different tasks in cognition, also influenced by personalized characteristics as lifestyle, demographics and psychometric measures including intelligence (Smith et al., 2015). Recently, functional and structural networks have been used to study the correlation between brain organization and intelligence. These studies revealed important correlations of local and widespread brain properties related to the cognitive functions and intelligence (Li et al., 2009; Douw et al., 2011). Global efficiency of functional brain networks and rich club organization appear to be important factors in intelligence (Van den Heuvel et al., 2009; Kim et al., 2016; Yeo et al., 2016). Moreover, small-world network organization has also been reported as a relevant feature to intelligence and neural network efficiency (Micheloyannis et al., 2006; Li et al., 2009) with observed differences between men and women (Douw et al., 2011). More differences related to the organization of brain networks across genders have been identified in default mode network, revealing local as well as widespread connection effects (Allen et al., 2011; Tomasi and Volkow, 2012; Szalkai et al., 2015).

Prefrontal and posterior parietal brain lobes, which are mostly related to intelligence (Basten et al., 2015; Ryman et al., 2016), have many connections to cerebellum (Koziol et al., 2014; Styliadis et al., 2015). Furthermore, there are many factors involved in cognitive processes justifying the examination of various brain areas in relation to IQ aspects, like the basal ganglia implicated in cognitive task processing. Even though it is known that the cerebellum is actively involved in cognitive processes (Koziol et al., 2014; Styliadis et al., 2015), there is a limited research investigating its relationship with IQ.

Considering all the above, the driving question of this study concerns the extent to which the cerebellum is related to intelligence, in men and women, beyond the cognitive processes. More specifically, we study the network organization in individual groups of different gender and IQ levels. For this reason, we constructed functional networks of the cerebellum using rs-fMRI data from individuals with high and low intelligence ratings. Then, we computed the corresponding Minimum Spanning Trees (MSTs) and compared them in order to identify significant local and widespread differences based on a variety of global and local network metrics. The MST is a widely used method that is able to preserve only the most important connections within a network without introducing threshold-related bias. As a result, it highlights only those edges that play a major role in the information transfer within the network. Using this strategy, our goal is to examine whether the MST topology can highlight significant differences among different IQ groups in the cerebellum. The fundamental hypothesis of this study is that the local and global characteristics of the cerebellar network exhibit significant differences which are related to gender and IQ.

## Materials and methods

### Subjects

Our data was collected from the Human Connectome Project (HCP) database, an open-source database aiming to provide deep examination of the human brain connectome (Van Essen et al., 2013). The HCP is the result of efforts of co-investigators from the University of California, Los Angeles, Martinos Center for Biomedical Imaging at Massachusetts General Hospital (MGH), Washington University, and the University of Minnesota. The present study analyzes rs-fMRI data collected from the HCP database after the HCP S500 + MEG2 data release, between the first six quarterly releases (Q1–Q6), with few cases also collected in Q7 and later. Functional magnetic resonance imaging (fMRI) data was initially acquired from 492 healthy subjects at rest with eyes open with relaxed fixation on a projected bright cross-hair on a dark background (and presented in a darkened room) (Van Essen et al., 2013). All subjects with psychiatric history, extensive substance use and hard alcohol history have been removed since the cerebellum is heavily impacted by alcohol abuse/dependence (Sullivan et al., 2010) and there is also evidence to suggest that the cerebellum is impacted by marijuana as well (Block et al., 2000; Lopez-Larson et al., 2011; Solowij et al., 2011). Moreover, additional information related to siblings and twins have been obtained. The population has been restricted to only one member of a sibling/twin pair so as to overcome shared variance issues. Fluid IQ scores were obtained per subject prior to scanning. Finally, subjects were separated based on their fluid IQ scores into two groups as described in the following section.

### IQ groups formation

Crystallized intelligence is conceptualized as the product of experience, both cultural and educational, in interaction with fluid intelligence, which implies the existence of an intersection between crystallized and fluid intelligence as far as the educational experience is (exclusively) concerned (Barch et al., 2013; Happé, 2013; Schipolowski et al., 2014). The HCP database provides fluid intelligence measures obtained using a Form-A of an abbreviated version of the Raven's patterns, developed by Gur and colleagues (Bilker et al., 2012; Barch et al., 2013). More specifically, participants were presented with patterns made up of 2 × 2, 3 × 3, or 5 × 5 arrangements of squares, with one of the squares missing. Each participant must pick one of five response choices that best fits the missing square on the pattern. The task has 24 items and 3 bonus items, arranged in order of increasing difficulty. However, the task discontinues if the participant makes 5 incorrect responses in a row. Median response times (MRTs) were also collected per subject in order to study associations with brain measures.

In this study, IQ score is defined as the number of correct responses per subject. The score distribution was found to be left-skewed (skewness = −0.49), implying that most of the subjects tend to answer correctly most of the items. In order to define the low and high-IQ groups, we first find the median of the IQ score distribution from all 492 subjects (approximately 16), as well as the lower quartile (approximately 6). The minimum score is 3 and the maximum score is 24. An IQ score of 3 is considered very low in practice and since only two subjects responded in this range, they were removed from further analysis without affecting the overall IQ distribution. We define the low-IQ score within the interval from 4 to 10 (median minus one quartile), whereas the upper IQ interval defines scores from 22 (median plus one quartile) to the maximum score of 24. As a result, the low-IQ group includes 69 subjects, whereas the high-IQ group is composed of 67 subjects in total. The mid-IQ subjects are discarded, so that our population of interest consists of 136 well-separated subjects (69 low-IQ/67 high-IQ). More specifically, there are 25 males and 44 females in the low-IQ group, while 29 males and 38 females are involved in the high-IQ group. As far as the educational experience is concerned, both the low-IQ and the high-IQ subjects had an average of approximately 10 years of educational experience although 12 low-IQ subjects and 8 high-IQ subjects were still respondent in school for degree courses. Ages are provided by the HCP database in 4 and 5-year intervals. Only 1 subject was older than 36 years (in high-IQ group), 26 subjects were between 22 and 25 years old (low/high-IQ; 17/9), 56 subjects were between 26 and 30 years old (low/high-IQ; 24/32) and finally 53 subjects were in the age range between 31 and 35 years (low/high-IQ; 28/25). Notice that these age intervals are not wide enough to support the consideration of age influences to intelligence (Li et al., 2004). It is worth mentioning that our population of interest consists of young and healthy adults that underwent several clinical examinations and the large number of estimated network parameters are satisfactory for subsequent statistical analyses.

### Resting state fMRI data

Resting-state BOLD fMRI data were obtained through a gradient-echo EPI sequence from a 3T scanner (91 volumes; *TR* = 720 ms, *TE* = 33.1 ms, *FA* = 52°, FOV = 208 × 180 mm, slice thickness = 2.0 mm; 2.0 mm isotropic voxels) (Van Essen et al., 2012, 2013). Pre-processed BOLD time-series (15 min duration, 1,200 frames) were acquired from the selected 136 subjects. HCP Investigators already performed straightforward pre-processing for de-noising the data using Independent Component Analysis (ICA) implemented on FSL's MELODIC tool (Beckmann and Smith, 2004). HCP investigators have also performed basic preprocessing pipelines. More specifically, two MR functional pipelines were applied (Glasser et al., 2013; Van Essen et al., 2013). The first volume-based pipeline removes spatial distortions, realigns volumes using FSL's FLIRT-based motion correction, normalizes the intensity of 4D images to a global mean, registers data into MNI space and finally masks the data with the final brain mask derived from FreeSurfer segmentation, while the second surface-based pipeline aims at transforming the time series from volume space to CIFTI gray-ordinates standard space with 2 mm average surface vertex and subcortical volume spacing. Surface data was smoothed using a 2 mm FWHM kernel and ICA was used to isolate independent components from the data. The components were then inserted into the FIX tool to preserve only information relevant components, which were used to reconstruct the de-noised signals. Further details on the preprocessing pipelines are provided in (Glasser et al., 2013; Van Essen et al., 2013).

### Cerebellum's anatomical parcellation process

**Figure 1:**
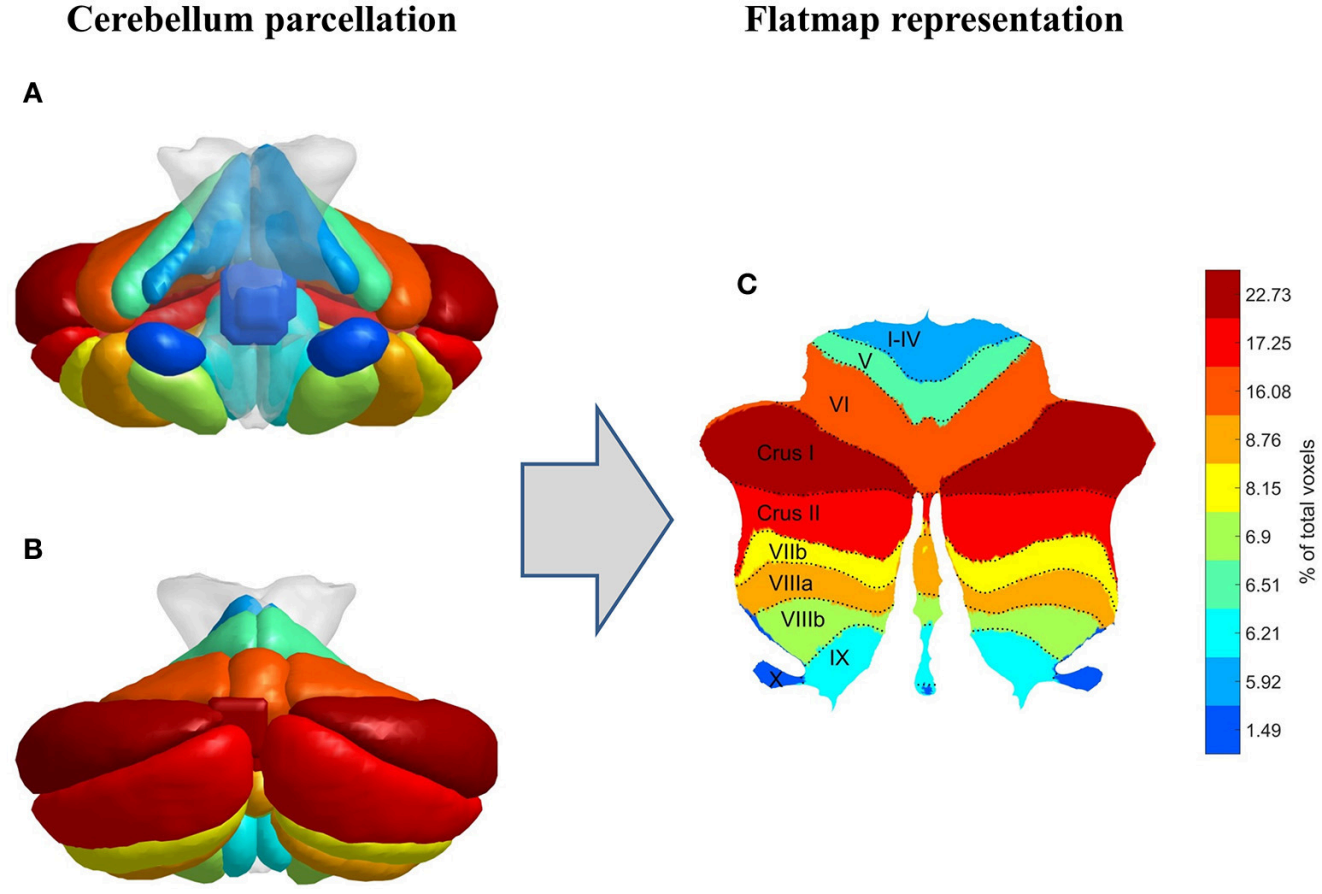
**Cerebellum parcellation procedure (coronal view**, **A**: front, **B**: back) followed by its flat surface representation **(C)**. Color coding is based on each lobule's volumetric size.

Based on the standard cerebellum anatomical atlas provided by the Spatially Unbiased Infratentorial Template (SUIT) toolbox (Diedrichsen et al., 2009; Diedrichsen and Zotow, 2015), the cerebellum was parcellated into 28 lobules or regions of interest (ROIs), which are classified as motor related (I–IV, V, VI), cognitive and emotional related (Crus I, Crus II, VIIb, VIIIa, VIIIb, IX, X) according to Stoodley and Schmahmann (2009), Stoodley et al. (2012), E et al. (2014), and Koziol et al. (2014), as shown in Figure 1. In order to avoid influences of intracranial volume differences among gender and IQ groups, all MRI structures were matched to the same model through the aforementioned parcellation procedure which was based on the standard (normalized) SUIT anatomical cerebellum atlas. The volume of each ROI was defined as the number of its voxels and was calculated from the standard SUIT cerebellum atlas (Diedrichsen et al., 2009; Diedrichsen and Zotow, 2015). As a result, the size of each ROI is common in all subjects, irrespective of IQ or gender factors. SUIT's standard cerebellum atlas was based on the hand-segmentation of 20 healthy young participants and was already registered on the MNI space (see Diedrichsen et al., 2009, for further information). In addition, a flat surface representation of the cerebellum according to Diedrichsen and Zotow (2015) is provided in Figure 1, where color coding has been applied based on each lobule's volumetric size. It is obvious from Figure 1 that lobule Crus I is the largest one including almost 23% of the total cerebellar volume, whereas lobule X is the smallest ROI including almost 1.5% of the total volume. Vermis Crus I contains less than 0.005% of the total cerebellar volume and is excluded from further analysis. The anatomical parcellation of cerebellum was performed for all 136 subjects separately, in order to extract the average BOLD signals from the remaining 27 ROIs, based on SUIT's standard cerebellum atlas.

### Graph analysis

#### Weighted—undirected graphs

According to Graph Theory, a graph *G* can be defined as a pair (*V, E*) where *V* is a set of nodes and *E* is a set of edges (Reijneveld et al., 2007; Fornito et al., 2013). Weighted and undirected graphs were constructed in this study. After the extraction of the BOLD time-series from cerebellum's parcellation procedure (Section Cerebellum's Anatomical Parcellation Process), the average BOLD time-series were computed per ROI and for every subject separately. Cerebellum's functional connectivity was then assessed by computing Pearson's correlation coefficients between each pair of the 27 ROIs inside the cerebellum, leading to a 27 × 27 correlation (adjacency) matrix per subject in both IQ groups. Negative correlations were discarded from further analysis (Bohr et al., 2013). The adjacency matrix is in fact a weighted and undirected graph with 27 nodes, which are designated as centers of mass on ROIs, with a maximum number of 351 edges per graph.

#### Small-world network topology

A small-world network architecture (Watts and Strogatz, 1998) combines high clustering coefficient and small characteristic path-length. In order to examine the small-world properties of the cerebellum network associated with the IQ groups, we should compare them to a null model. In order to produce this null model, a total of 100 random weighted and undirected graphs (edge and weight preserving) were formed for computing ${C^{w}}_{rand}$ and ${L^{w}}_{rand}$ using Brain Connectivity Toolbox (Rubinov and Sporns, 2010). In this study, the weighted versions of clustering coefficient and characteristic path-length were used in order to compute the small-worldness index (Stam et al., 2009; Rubinov and Sporns, 2010; Otte et al., 2015), as presented in Table 1. In fact, a small-world network is characterized by higher segregation (γ^*w*^ ≫ 1) than a random network and almost equal integration (λ^*w*^ ≈ 1) with that of a random network and therefore achieves a small-worldness index larger than 1 (Van den Heuvel et al., 2008, 2009; Stam et al., 2014). This network architecture manages to achieve efficient information transfer at low wiring cost.

**Table 1:** **Network descriptors used in this study**.

**Symbol**	**Interpretation**	**Mathematical expression**	**Implication**
*G*	Graph	–	Weighted and undirected graph
*V*	Set of vertices	–	Set of n-nodes
*E*	Set of edges	–	Set of n^*^(n−1)/2 maximum edges
*N*_*leaf*_	Leaf nodes	–	Number of nodes with degree equal to one
*w*_*ij*_	Weight	–	Weight of the edge connecting nodes *i* and *j*
$t_{i}^{w}$	Number of triangles	$t_{i}^{w}=\frac{1}{2}{\sum_{j,k\in V}\left(w_{ij}w_{jk}w_{kl}\right)}^{1/3}$	Weighted geometric mean of triangles around a node
$d_{ij}^{w}$	Shortest path	-	Shortest weighted path between nodes *i* and *j*
$C_{i}^{w}$	Weighted clustering coefficient	$C_{i}^{w}=\sum_{i\in G}\frac{2t_{i}^{w}}{k_{i}(k_{i}-1)}$	Segregation measure that quantifies the local connectedness of a network
*C*_*w*_	Average weighted clustering coefficient	$C^{w}=\frac{1}{n}\sum_{i\in G}C_{i}^{w}$	A global version of the weighted clustering coefficient used for computing σ^*w*^
*L*_*w*_	Weighted characteristic path length	$L^{w}=\frac{1}{n}\sum_{i\in G,j\neq i}\frac{d_{ij}^{w}}{n-1}$	Integration measure
γ^*w*^	Gamma	$\gamma^{w}=C^{w}/{C^{w}}_{rand}$	Ratio of the weighted clustering coefficients between original and random networks
λ^*w*^	Lambda	$\lambda^{w}=L^{w}/{L^{w}}_{rand}$	Ratio of weighted path lengths between original and random networks
σ^*w*^	Small-worldness index	σ^*w*^ = γ^*w*^/λ^*w*^	Reveals whether a network has an optimal organization or not
*conn*	Connectivity	$conn=\frac{1}{n(n-1)}\cdot \sum_{\begin{array}{c} w_{ij}\in G\\ i\neq j \end{array}}w_{i,j}$	Measures the connectedness of a network in terms of network's density, where *p*_*kl*_ is the number of shortest paths between nodes *k* and *l* and $p_{kl}^{j}$ is the number of shortest paths between *k* and *l* that pass through node *j*
*k*	Degree	$k_{i}=\sum_{j\in V}a_{ij}$	Number of neighbors connected to a node (hub metric)
*BC*	Betweenness centrality	$BC_{i}=\sum_{\begin{array}{c} k,l\in V\\ k\neq l,\;k\neq i,\;l\neq i \end{array}}\frac{p_{kl}^{i}}{p_{kl}}$	Quantifies the importance of a node (hub metric)
*ECC*	Eccentricity	–	Indicates whether a node is central or peripheral in a network
*d*	Diameter	–	Maximum eccentricity
*r*	Radius	–	Minimum eccentricity
*L*_*f*_	Leaf fraction	*L*_*f*_ = *N*_*leaf*_/*n*−1	Fraction of nodes with degree equal to one
*T*_*h*_	Tree-hierarchy	$T_{h}=\frac{N_{leaf}}{2\left(n-1\right)BC_{max}}$	Quantifies the balance between diameter reduction and overload prevention
κ	Kappa or degree divergence	$\kappa =\frac{\langle k2\rangle }{\langle k\rangle }$	Measure of the broadness of the degree distribution
*r*_*deg*_	Degree correlation	–	Quantifies the influence of a node's degree by its neighbors

#### Minimum spanning trees

Toward the characterization of a graph's architecture, it would be convenient to summarize it with a structure that (i) overcomes biases introduced by comparing networks with different number of edges and (ii) eliminates any disconnected syndromes within the network (for further network-comparison issues see Stam and van Straaten, 2012; van Diessen et al., 2015). A straightforward approach that fulfills these specifications operates on the concept of MSTs, a widespread graph analysis method recently employed for brain functional-connectivity assessment (Tewarie et al., 2014, 2015a; Van Diessen et al., 2014; Otte et al., 2015; Van Dellen et al., 2015; Stam et al., 2016). A spanning tree is a connected subgraph of the original graph with n-nodes and exactly n−1 edges (Stam et al., 2014; Tewarie et al., 2015b). A MST is a spanning tree that manages to preserve only the edges that minimize the total cost defined as the sum of the weights of the edges. In our study, MSTs were constructed using Kruskal's algorithm (Kruskal, 1956). The algorithm begins with n-disconnected nodes and orders the weights in ascending order. Afterwards, the edge with the smallest weight is selected to connect two nodes, unless the selected edge creates a loop. The above procedure is repeated until a loopless subgraph with n-nodes and n−1 edges is constructed. In order to preserve the strongest connections within the original network, Kruskal's algorithm is executed so as to minimize the inverse of the total cost and, thus, retain only edges that maximize the total cost (Boersma et al., 2013; Tewarie et al., 2015a; Van Dellen et al., 2015). The result is an acyclic subgraph of the original graph that manages to preserve only the strongest connections (edges). The MST provides a graph representation that absorbs population characteristics into a compact form and facilitates the distinction of different populations through the computation of various metrics or descriptors. Recall that such MST comparisons assess the effects of only the strongest connections within the original network topology and therefore the MST can be suitably used to examine the IQ's effect in cerebellum.

#### Local and global MST descriptors

Three local and six global MST metrics were computed in order to describe the topological characteristics of each MST (Stam and van Straaten, 2012; Otte et al., 2015; Tewarie et al., 2015a). The local MST descriptors are computed per node and normalized with their corresponding maximum values for appropriate comparisons. These metrics are summarized in Table 1. In particular, degree (DEG) is defined as the number of edges connected to a specific node, while betweenness centrality (BC) defines the fraction of all shortest paths in the network that pass through a specific node. Consequently, these two metrics can be used as hub indicators, since they provide useful information concerning the information flow within the network. Finally, eccentricity (ECC) is the longest shortest path from a particular node to any other node in the network. The global MST measures are defined on the basis of the entire network. Based on eccentricity's definition, diameter (*d*) is defined as the longest shortest path in the whole network, so that small diameter values denote better network cohesion, whereas radius (*r*) is defined as the smallest shortest path in the network. Leaf fraction (*L*_*f*_) is the fraction of leaf nodes in the network. Tree-hierarchy (*T*_*h*_) is a metric first introduced by Boersma et al. (2013) as an optimal tree configuration quantifier. An optimal tree is characterized by diameter reduction and overload prevention (small BC values), with the value of *T*_*h*_ approximating 0.5. Kappa (κ) or degree divergence is mainly related to the synchronization level of tree nodes. Finally, degree correlation (*r*_*deg*_) is computed through the Pearson correlation coefficient of the degrees of pair of vertices connected by an edge (Boersma et al., 2013).

#### Hub(s) detection

Nodes with high BC and DEG values are characterized as critical nodes (hubs) and are used to determine the information flow within the network. In order to specify hub nodes for a group population, we computed the percentage of maximum BC, DEG values in every ROI, for low/high-IQ males/females.

### Statistical analysis

Small-world properties were investigated for all weighted and undirected graphs and, afterwards, the corresponding MSTs were constructed. Subsequently, three local (BC, ECC, DEG) and six global (diameter, degree correlation, radius, kappa, leaf fraction, tree hierarchy) metrics were computed in order to examine the topological and functional characteristics of every MST. Moreover, several global weighted graph metrics, including average weighted clustering coefficient, characteristic path length, small-worldness and connectivity, were also examined in our procedure. The feature datasets are non-normally distributed, in general, so that natural log-transformation was applied in order to approximate normal distribution properties, with the addition of a very small constant (1· e−24) for avoiding zero-value transforms. Statistical analysis was performed using 1-way unbalanced ANOVA. In total, five cases were investigated in order to test for differences between (i) low and high-IQ groups (low/high-IQ; 69/67), (ii) males in low and high-IQ groups (low/high-IQ; 25/29), (iii) females in low and high-IQ groups (low/high-IQ; 44/38), (iv) males and females in low-IQ group (males/females; 25/44), (v) males and females in high-IQ group (males/females; 29/38). All *p*-values were corrected based on False Discovery Rate (FDR) using the Benjamini-Hochberg procedure (Benjamini and Hochberg, 1995) with the significance level set to 0.05.

## Results

### Small-world network structure

Cerebellum manifests a small-world network structure in both low and high-IQ populations (low-IQ: 1.2644 ± 0.1765; high-IQ: 1.2126 ± 0.1010), implying that cerebellum network works efficiently at low wiring cost for both IQ groups. τhe same evidence stands for males/females comparisons (low-IQ males: 1.2334 ± 0.1243; high-IQ males: 1.2287 ± 0.1243; low-IQ females: 1.2821 ± 0.1994; high-IQ females: 1.2002 ± 0.0783). Low-IQ subjects tend to have higher average clustering coefficient (1.1939 ± 0.0857) but smaller characteristic path length (0.9548 ± 0.0917) than their high-IQ peers (avg. clustering coefficient: 1.1634 ± 0.0564; characteristic path length: 0.9640 ± 0.0689). Moreover, low-IQ males and females have similar characteristic path lengths (low-IQ males: 0.9523 ± 0.0774; low-IQ females: 0.9562 ± 0.0997) but females have higher average clustering coefficient (low-IQ males: 1.1671 ± 0.0661; low-IQ females: 1.2092 ± 0.0923). In addition, high-IQ females have higher average clustering coefficient than high-IQ males (high-IQ males: 1.1520 ± 0.0396; high-IQ females: 1.1720 ± 0.0657), as well as characteristic path-length (high-IQ males: 0.9454 ± 0.0878; high-IQ females: 0.9781 ± 0.0466). The above results are summarized on Supplementary Table 1. Statistical analysis results on these measures as well as on the rest MST measures are presented later on, in Sections Differences between Low and High-IQ Groups and Differences between Low and High-IQ Groups Per Gender.

### MSTs in low and high-IQ subjects

**Figure 2:**
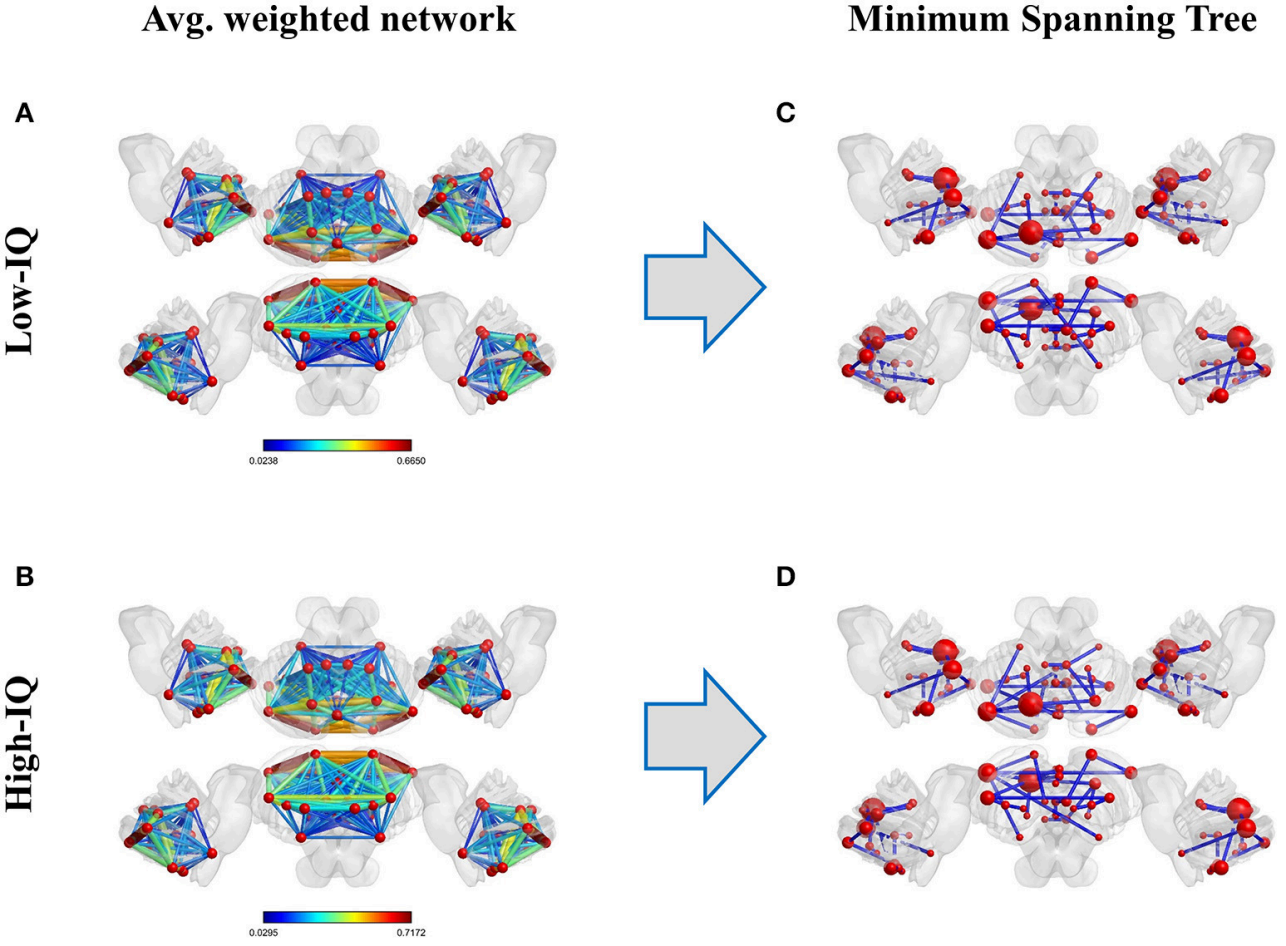
**Average weighted and undirected graphs per IQ group (left panel**, **A**: low-IQ and **B**: high-IQ) and their corresponding MSTs (right panel, **C**: low-IQ and **D**: high-IQ). On the latter representation, each node's size depends linearly on its average BC value.

**Figure 3:**
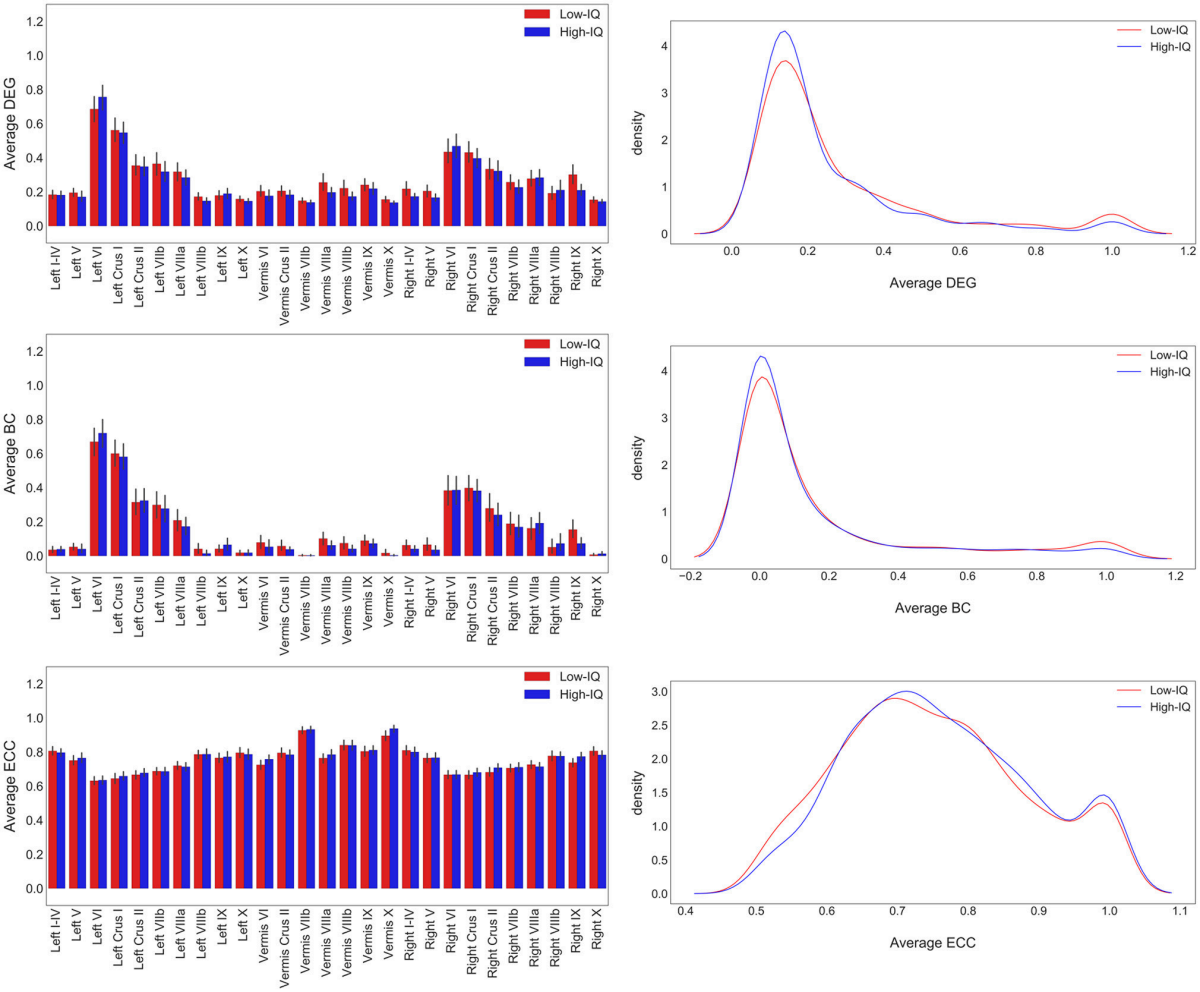
**Average DEG, BC, ECC values per ROI for both IQ groups on the left panel and the corresponding distributions on the right panel**. DEG and BC values tend to have similar distributions since the number of connections that pass through a specific node is related with the overloadness within the network and vice versa. The number of nodes with the highest BC and DEG values (hubs) is small. On the other hand, ECC values exhibit a much more homogeneous diffuse. Nodes with small eccentricity values are much closer to the center of the network and are characterized by higher BC and DEG values.

MSTs were computed for both low and high-IQ subjects, as described in Section Minimum Spanning Trees. The average weighted and undirected graphs and the resulting MSTs are presented in Figure 2 for illustration purposes only, using BrainNet viewer (Xia et al., 2013). Although the networks in low and high-IQ populations seem similar, their differences are revealed by the aforementioned metrics that quantify the network's topological structure. The average DEG, BC, ECC values for low and high-IQ groups are displayed in Figure 3 and analyzed in more detail in Supplementary Tables 2–4, alongside with the average MST local metrics for low/high-IQ males and females.

### Hubs

**Figure 4:**
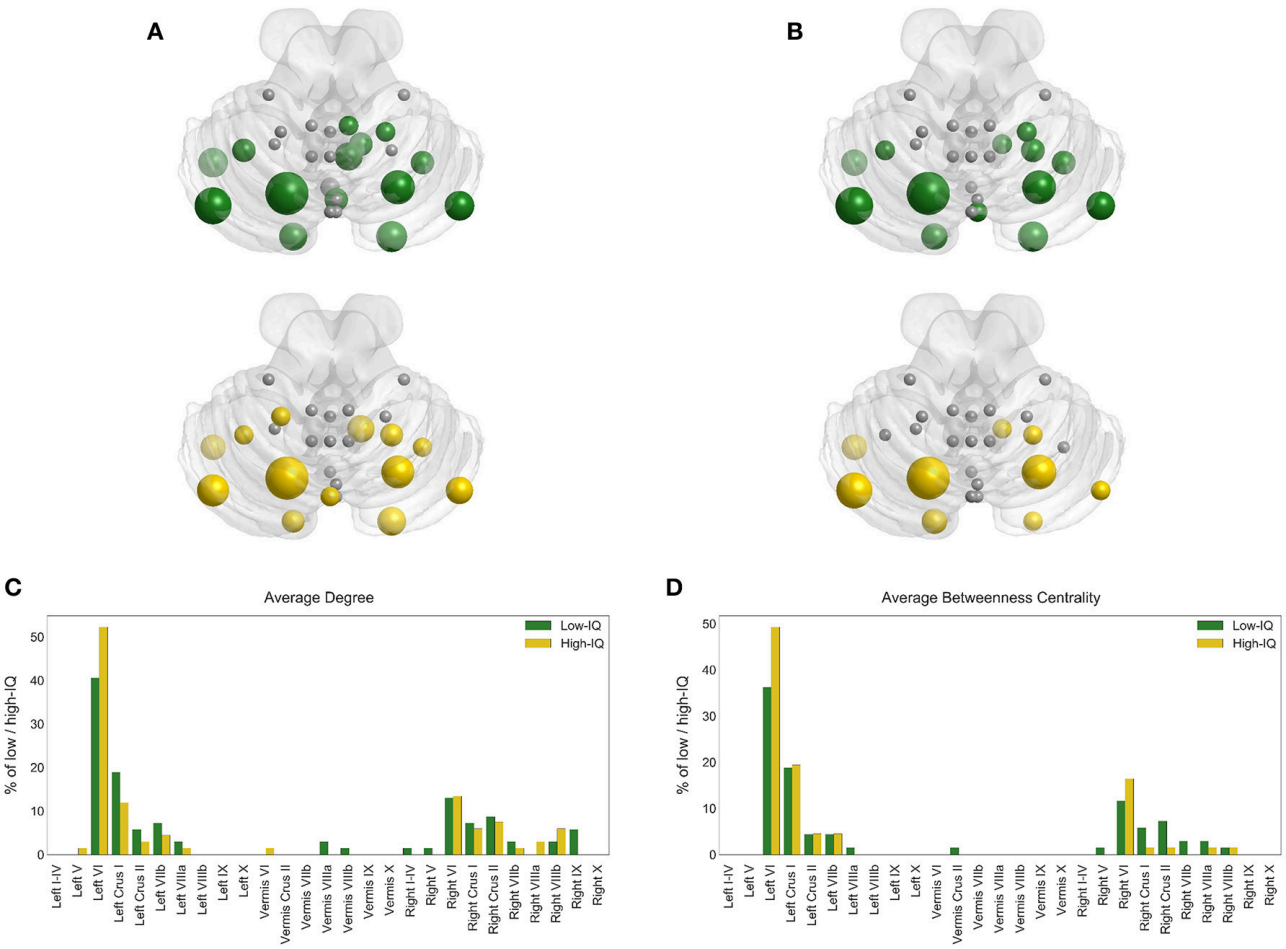
**Hub locations on cerebellum for low (green) and high (yellow) IQ groups based on BC (A)** and DEG **(B)**. The size of each node depends on the percentage of low/high-IQ subjects with the highest BC **(C)** and DEG **(D)** values.

Hub analysis reveals that lobule Left VI is a critical node having the highest BC value in almost 36% of the low-IQ population and 49% of the high-IQ population, as well as the highest DEG value in 41% of the low-IQ population and 52% of the high-IQ population (Figure 4). Thus, lobule Left VI is responsible for “traffic” monitoring in the cerebellum network for both IQ groups. This Left VI's significance in information transfer is of the same importance for low and high-IQ subjects, but with a higher manifestation in the high-IQ population. Moreover, lobules Left Crus I and Right VI can also be characterized as hubs, but with a smaller dominance than Left VI. The Left Crus I lobule activates more in the low-IQ population, as indicated by DEG and almost equally activated for both groups as indicated by BC. Alternatively, the Right VI lobule is more active in the high-IQ population, even though to a smaller extent than other lobules.

These hub indications have also been validated based on sex for low and high-IQ male/female populations. Region Left VI is indeed a critical node for all groups, having the highest BC value in 40% of low-IQ males and 34% of low-IQ females, as well as the highest DEG value in 48% of low-IQ males and 36% of low-IQ females (Supplementary Figure 1). In the high-IQ population, Left VI demonstrates the highest BC value in 48% of high-IQ males and 50% of high-IQ females, as well as the highest DEG value in 52% of high-IQ males and 53% of high-IQ females (Supplementary Figure 2). In addition, the Left VI hub appears stronger in high-IQ females than high-IQ males. Left Crus I is more activated in low/high-IQ males as indicated by both BC and DEG measures whereas the opposite stands for Right VI which appears to be more activated in low/high-IQ females. In each individual figure we can compare the size and the number of nodes that participate in hub analysis. The bar plots (on the lower panel) and the cerebellar anatomical plots (on the upper panel) encode the same information but offer additional visual interpretation on the cerebellar surface, thus providing the anatomical location for each hub. These figures offer a clear representation concerning the hub locations for the different populations of interest.

### Correlation between DEG, BC, and median response times

In order to examine which region interacts the most with the Median Response Time (MRT), we computed Pearson's correlation coefficients (per subject) between each ROI's DEG, BC measures (hub indicators) and subjects MRT values by taking into consideration the IQ factor. Then, the region with the highest correlation was selected.

**Figure 5:**
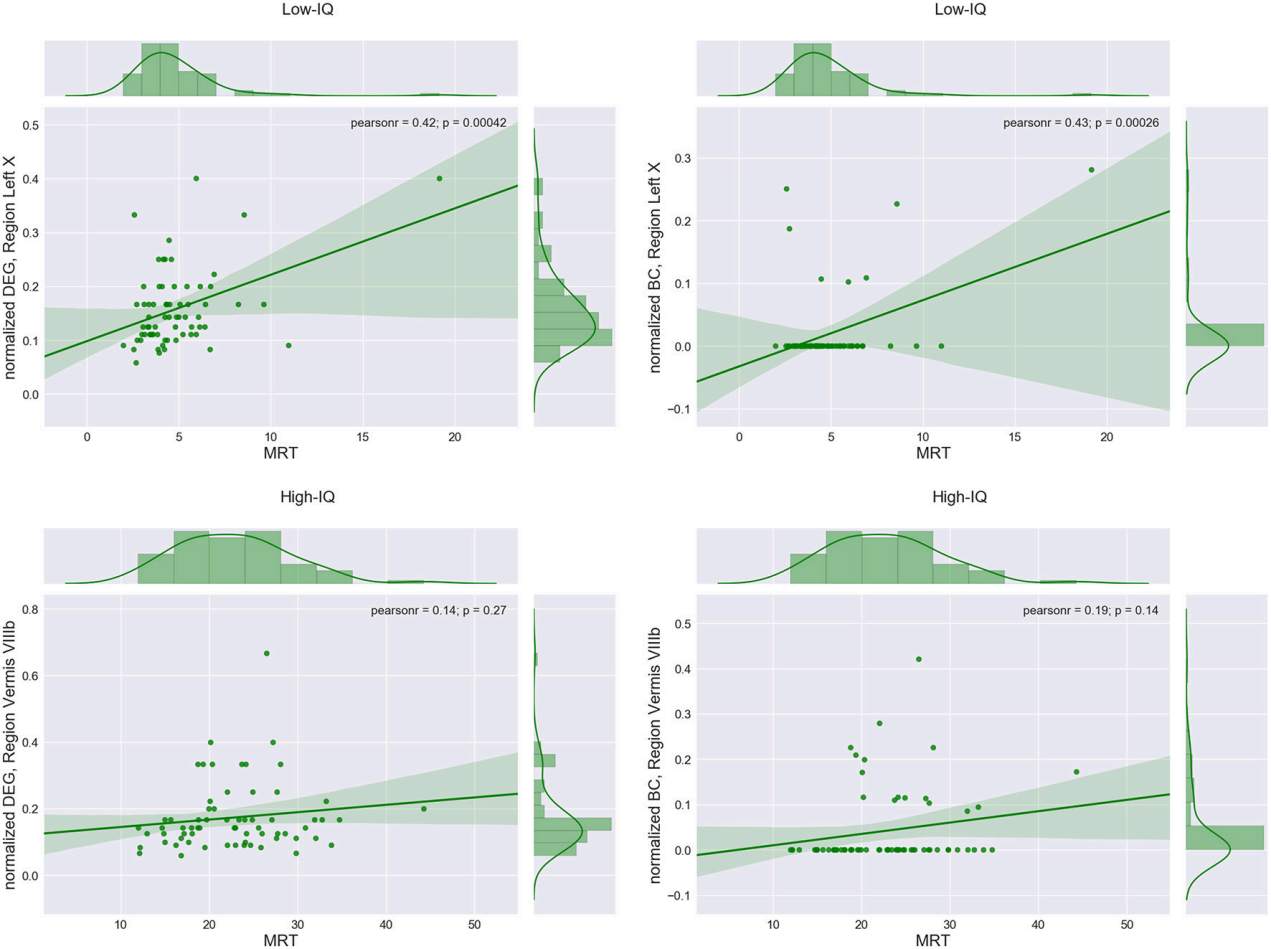
**Regions with the maximum correlation between average DEG or BC measure and median response times (MRTs) for low and high-IQ groups**.

Region Left X exhibited the highest positive significant correlation between DEG and MRT for the low-IQ group (*r* = 0.42, *p* = 0.0004) as well as between BC and MRT (*r* = 0.43, *p* = 0.0003). On the other hand, region Vermis VIIIb indicated the highest positive correlation between DEG and MRT for the high-IQ group (*r* = 0.14, *p* = 0.27) as well as between BC and MRT (*r* = 0.19, *p* = 0.14), without however any statistical significance at all (Figure 5, Table 2).

**Table 2:** **ROI(s) with the maximum correlation coefficient between MRT and DEG or BC measure for both IQ groups and gender**.

	**Group**	**DEG**			**BC**		
		**Maximum correlation coefficient**	***p***	**ROI**	**Maximum correlation coefficient**	***p***	**ROI**
Low IQ	Total	0.42	**0.0004**	Left X	0.43	**0.0003**	Left X
	Males	0.57	**0.0034**	Left Crus II	0.54	**0.006**	Vermis VIIIb
	Females	0.47	**0.0014**	Left X	0.46	**0.002**	Left X
High IQ	Total	0.14	0.27	Vermis VIIIb	0.19	0.14	Vermis VIIIb
	Males	0.21	0.29	Left VI	0.25	0.2	Right X
	Females	0.23	0.18	Vermis VIIIb	0.2	0.24	Vermis VIIIb

*With bold highlight: statistical significant results (p < 0.05)*.

The same procedure was repeated for males and females in both IQ groups. In the male population, Left Crus II exhibited the highest positive significant correlation between DEG and MRT for the low-IQ group (*r* = 0.57, *p* = 0.0034), whereas region Left VI was the one for the high-IQ group (*r* = 0.21, *p* = 0.29) but without any significance. Alternatively, the lobule with the highest positive significant correlation between BC and MRT was Vermis VIIIb (*r* = 0.54, *p* = 0.006) for the low-IQ group while Right X was selected for the high-IQ group (*r* = 0.25, *p* = 0.2) without again any significance (Supplementary Figure 3, Table 2). Focusing now on females, region Left X exhibited the highest positive significant correlation between DEG and MRT for the low-IQ group (*r* = 0.47, *p* = 0.0014). On the other hand, region Vermis VIIIb was the one with the highest positive (non-significant) correlation for the high-IQ group (*r* = 0.23, *p* = 0.18). Finally, the region with the highest correlation between BC and MRT was again Left X (*r* = 0.46, *p* = 0.002) for the low-IQ group and Vermis VIIIb for the high-IQ group (*r* = 0.2, *p* = 0.24) but without any significance (Supplementary Figure 4, Table 2). In general, all regions that exhibited the highest correlations between DEG/BC and MRT, in the high-IQ group, were non-significant.

### Differences between low and high-IQ groups

Local MST metrics did not exhibit any significant differences between low and high-IQ groups (not shown). On the other hand, significant differences were found in four global metrics (Supplementary Table 5). In particular, these differences are reflected for the metrics of average clustering coefficient (low-IQ: 1.1939 ± 0.0857; high-IQ: 1.1634 ± 0.0564) (*F* = 5.8769, *p* = 0.0167), connectivity (low-IQ: 0.1784 ± 0.0763; high-IQ: 0.2073 ± 0.0878) (*F* = 5.1324, *p* = 0.0251), diameter (low-IQ: 0.4002 ± 0.1632; high-IQ: 0.3376 ± 0.1215) (*F* = 5.2927, *p* = 0.0230) and radius (low-IQ: 0.4101 ± 0.1641; high-IQ: 0.3540 ± 0.1400) (*F* = 4.3788, *p* = 0.0383).

### Differences between low and high-IQ groups per gender

Specializing the comparisons per gender population, local MST metrics did not exhibit any significant differences between low/high-IQ males or females (not shown). In addition, no significant differences were found on any global metrics between low and high-IQ males (Supplementary Table 6). However, significant differences were identified between low and high-IQ females by five global metrics (Table 3), specifically for average clustering coefficient (low-IQ females: 1.2092 ± 0.0923; high-IQ females: 1.1720 ± 0.0657) (*F* = 4.2866, *p* = 0.0416), small-worldness (low-IQ females: 1.2821 ± 0.1994; high-IQ females: 1.2002 ± 0.0783) (*F* = 4.8060, *p* = 0.0313), connectivity (low-IQ females: 0.1629 ± 0.0689; high-IQ females: 0.2014 ± 0.0856) (*F* = 5.8085, *p* = 0.0182), diameter (low-IQ females: 0.4291 ± 0.1654; high-IQ: 0.3450 ± 0.1263) (*F* = 6.8101, *p* = 0.0108) and radius (low-IQ females: 0.4394 ± 0.1648; high-IQ females: 0.3629 ± 0.1510) (*F* = 5.8233, *p* = 0.0181).

**Table 3:** **Statistical analysis results per female IQ group and low IQ group for the main network metrics**.

**Metric**	**Low-IQ females**	**High-IQ females**	***F***	***P***	**Low-IQ males**	**Low-IQ females**	***F***	***P***
	**Mean ± SD**	**Mean ± SD**			**Mean ± SD**	**Mean ± SD**		
*C*_*w*_	1.2092 ± 0.0923	1.1720 ± 0.0657	4.2866	**0.0416**	1.1671 ± 0.0661	1.2092 ± 0.0923	4.1227	**0.0463**
*L*_*w*_	0.9562 ± 0.0997	0.9781 ± 0.0466	2.1312	0.1482	0.9523 ± 0.0774	0.9562 ± 0.0997	0.0055	0.9412
σ^*w*^	1.2821 ± 0.1994	1.2002 ± 0.0783	4.8060	**0.0313**	1.2334 ± 0.1243	1.2821 ± 0.1994	0.9492	0.3334
*conn*	0.1629 ± 0.0689	0.2014 ± 0.0856	5.8085	**0.0182**	0.2058 ± 0.0822	0.1629 ± 0.0689	4.7494	**0.0328**
*d*	0.4291 ± 0.1654	0.3450 ± 0.1263	6.8101	**0.0108**	0.3493 ± 0.1491	0.4291 ± 0.1654	5.1985	**0.0258**
*r*	0.4394 ± 0.1648	0.3629 ± 0.1510	5.8233	**0.0181**	0.3584 ± 0.1524	0.4394 ± 0.1648	5.3445	**0.0239**
*L*_*f*_	0.5935 ± 0.1034	0.6063 ± 0.0940	0.4147	0.5214	0.5892 ± 0.0775	0.5935 ± 0.1034	0.0001	0.9907
*T*_*h*_	0.2968 ± 0.0517	0.3031 ± 0.0470	0.4147	0.5214	0.2946 ± 0.0387	0.2968 ± 0.0517	0.0001	0.9907
κ	2.2465 ± 0.2827	2.3164 ± 0.2666	1.4271	0.2358	2.3391 ± 0.4098	2.2465 ± 0.2827	0.7141	0.4011
*r*_*deg*_	−0.3474 ± 0.1338	−0.3766 ± 0.1264	1.6282	0.2056	−0.3374 ± 0.1138	−0.3474 ± 0.1338	0.0033	0.9544

*With bold highlight: statistical significant results (p < 0.05)*.

Four global metrics indicated significant differences between males and females in the low-IQ group (Table 3), specifically for average clustering coefficient (low-IQ males: 1.1671 ± 0.0661; low-IQ females: 1.2092 ± 0.0923) (*F* = 4.1227, *p* = 0.0463), connectivity (low-IQ males: 0.2058 ± 0.0822; low-IQ females: 0.1629 ± 0.0689) (*F* = 4.7494, *p* = 0.0328), diameter (low-IQ males: 0.3493 ± 0.1491; low-IQ females: 0.4291 ± 0.1654) (*F* = 5.1985, *p* = 0.0258) and radius (low-IQ males: 0.3584 ± 0.1524; low-IQ females: 0.4394 ± 0.1648) (*F* = 5.3445, *p* = 0.0239). Finally, one significant difference was found between high-IQ males and females (Supplementary Table 7) in characteristic path length (high-IQ males: 0.9454 ± 0.0878; high-IQ females: 0.9781 ± 0.046) (*F* = 4.5376, *p* = 0.0369).

In summary, all three local MST metrics (DEG, BC, ECC) did not exhibit any significant differences among low/high-IQ groups as well as between the four possible gender-based group combinations (low/high-IQ males, low/high-IQ females, low-IQ males/females, high-IQ males/females). On the other hand, four global metrics (average clustering coefficient, connectivity, diameter and radius) revealed significant differences between low and high-IQ groups as well as between low-IQ male and female populations. The same conclusion stands for low/high-IQ females, with the addition of the small-world metric as well. Characteristic path length was the only metric that exhibited significant difference between high-IQ males and females. As far as the low/high-IQ males are concerned, no significant differences were identified. Our findings in men are in a similar direction with respect to the IQ level, but appear not significant. Both sexes have the characteristics of small-world networks with differences in females indicative of higher cerebellar neural efficiency, especially in higher-IQ females. In relation to the activities of the lobules, the metrics of DEG, BC, and ECC showed no differences between low and high-IQ individuals, or between men and women. The ECC values showed a relative homogeneous diffuse distribution, indicative of a rather compact organization of the activity of the cerebellar lobules.

## Discussion and conclusions

To assess the hypothesis that the cerebellar functional networks at rest differ between low and high-IQ individuals and/or between men and women, we employed tools from network theory and analyzed fMRI networks at rest. The results were indicative of local and widespread differences of the functional organization, revealing differences in the importance of several cerebellar lobules and widespread network parameters.

The small-world network structure, characterized by high global and local efficiency, is a property of anatomical and functional brain networks. This configuration maximizes the efficiency and minimizes the costs of information processing. It implies high clustering of nodes (compatible with segregated or modular processing) and short path length (compatible with distributed or integrated processing) (Watts and Strogatz, 1998; Bassett and Bullmore, 2006) and has been extensively reported in EEG, MEG, Tractography and fMRI studies (Stam, 2004; Tewarie et al., 2014; Stam et al., 2016). Focusing on cerebellum, we confirmed this optimum organization using fMRI at rest (Supplementary Table 1). In females, small-worldness exhibited lower values in high-IQ individuals. The higher small-world organization, with higher clustering coefficient and lower path length in low-IQ females, is indicative of a more optimum functionally organized segregation and integration. In contrast, in high IQ females, the segregation and integration of the functional networks at rest can be explained as the idle state of more efficient reactivity in cognitive tasks, in accordance to the neural efficiency hypothesis. The same stands for the male population but without any significance. The neural efficiency hypothesis becomes relevant during brain activations, where more efficient individuals show lower brain activation as they functionally react easier. In agreement to these results, we found earlier that highly educated individuals showed less prominent small-world structure than their less educated and lower IQ counterparts (Micheloyannis et al., 2006).

It is expected that the intrinsic organization of cerebellar functions at rest follows the functional organization of the cerebrum so similar co-activation with the brain structures is expected (Liao et al., 2010; Kelly et al., 2012). Thus, differences between men and women, as well as between low and high-IQ individuals, which appear intrinsically in the cerebellar network organization at rest, are expected to reflect differences in cognitive functions in association with intelligence. It is interesting that our study supports these assumptions at a statistically significant level only in women. There is a trend toward the same direction in men, but without significant differences between low and high-IQ individuals. This differentiation between men and women is indicative of gender differences in cognitive functions which are associated with intelligence. In addition, the lobules with the highest DEG, BC values can be related mainly with cognitive functions, where a left cerebellar dominance is observed. An additional finding related to nodes of the cerebellum is that regions Left Crus II, Left X, and Vermis VIII in low-IQ individuals (both men and women) showed maximum correlation with the median response time, implying that these lobules become more important only in low-IQ individuals (Table 2). It is further known that these lobules are related to motor and cognitive functions (Koziol et al., 2014).

Additional differences between low and high-IQ women were found in some global parameters (Supplementary Table 7). The network connectivity was higher in high-IQ women than low-IQ women, with smaller diameter and radius values (Table 3). These findings show that the network organization in women with high-IQ at rest is more efficient. In combination with the findings of small-world organization, it may also signify the expression of neuronal-network efficiency in this sub-population. Male groups exhibit similar trends, but without any significance. The increased readiness and efficiency of network organization, as well as the lower small-worldness in high-IQ females, compared to low-IQ females and to men counterparts, forms an interesting finding of our study. According to the intrinsic cerebellar connections, these findings could be related to the fact that the cerebellar-cerebral coordination differs among individuals, with known language dominance in women, which is more effective in highly intelligent individuals. At this point, it is worth mentioning that the cerebellum receives multiple inputs from contra- and ipsilateral hemispheres (Suzuki et al., 2012; Sokolov et al., 2014). In particular, there is evident functional connectivity among mentalizing areas of the cerebrum (mainly medial prefrontal cortex, medial parietal cortex, and bilateral temporo-parietal region) and mentalizing areas of the cerebellum (mainly the posterior lateral cerebellar lobules) (Van Overwalle et al., 2015; Van Overwalle and Mariën, 2016).

In terms of DEG and BC metrics, many lobules exhibited higher values on the left side whereas some other lobules express right activation related to motor and cognitive functions but to a smaller extent; i.e., IV, V, VI and parts of HVIIb and HVIII related to motor function (Stoodley et al., 2012), while Crus I, Crus II, lobule VI, VIIa and VIIb related to cognitive function (Bernard et al., 2012). The dominance of DEG and BC on left lobules was exhibited in both sexes, but the stronger Left VI hub indication in high-IQ women is a novel finding and goes in parallel with other higher-level organizations in this group. The aforementioned hubs are related to frontal, pre-frontal, temporal, parietal lobes (lobule VI), frontal gyrus, precuneus, angular gyrus, interior parietal lobe (Crus I) (Bernard et al., 2012; Koziol et al., 2014).

Summarizing, the study of low and high-IQ individuals revealed that both sexes have the characteristics of small-world networks with differences in females indicative of higher neural efficiency of the cerebellum, especially in higher-IQ females. The more efficient network organization in women reflects the different hemispheric organization between genders. The considerations of three global metrics in women support this conclusion. Our findings in men are in a similar direction with respect to the IQ level, but appear not significant. The lower small-worldness in high-IQ females, compared to low-IQ females and to men counterparts, forms an interesting finding of our study. In addition, five global metrics (i.e., average clustering coefficient, small-worldness, connectivity, diameter and radius) revealed significant differences between low and high-IQ individuals, as well as within females in low and high-IQ groups. Three cerebellar lobules (i.e., Left Crus II, Left X, and Vermis VIII) in low-IQ individuals (both genders) showed maximum correlation with the median response time, implying increased effort dedicated locally by this population in cognitive tasks. One known difference between men and women is related to the dominance of the women hemispheres in language (van Dun et al., 2016). Additional anatomical differences between men and women have been demonstrated in several studies. In particular, although there is no difference in intelligence ability, the neural substrates of general intelligence are different between the sexes (Stam, 2004; Malpas et al., 2016). Moreover, the cerebellar functional connections depend on the IQ level, which is in accordance to the neural efficiency hypothesis. Future studies need to be addressed in order to clarify such differences in cerebellum-cerebral connections. The present findings combined with future studies could practically contribute to the examination of disturbances in cerebellum and/or cerebellar-cerebrum connections with respect to intelligence in both sexes.

## Ethics statement

We requested and received access to data collected by the Human Connectome Project (HCP) for the purpose of scientific investigation and agreed to their open access terms of use. The HCP consent procedure was approved by the Washington University institutional review board. For more information see Van Essen et al. (2013).

## Author contributions

VP executed the functional connectivity and statistical analysis of the data, prepared the figures and contributed to the writing of the Materials and Methods and Results. SM prepared the Introduction and interpreted the findings in the Discussion. MZ and MK had major contribution to the structuring of material and the detailed review of the manuscript.

### Conflict of interest statement

The authors declare that the research was conducted in the absence of any commercial or financial relationships that could be construed as a potential conflict of interest.

## Supplementary material

Supplementary Figure 1 **Hub locations on cerebellum for low-IQ males (light blue) and females (orange) based on BC (A)** and DEG **(B)**. The size of each node depends on the percentage of low-IQ males and females with the highest BC **(C)** and DEG **(D)** values, respectively.

Supplementary Figure 2 **Hub locations on cerebellum for high-IQ males (blue) and females (red) based on BC (A)** and DEG **(B)**. The size of each node depends on the percentage of high-IQ males and females with the highest BC **(C)** and DEG **(D)** values, respectively.

Supplementary Figure 3 **Regions with the maximum correlation between average DEG or BC measure and median response times (MRTs) for low and high-IQ males**.

Supplementary Figure 4 **Regions with the maximum correlation between average DEG or BC measure and median response times (MRTs) for low and high-IQ females**.

Supplementary Table 1 **Small-world properties information**.

Supplementary Table 2 **Mean ± SD values based on IQ, for the normalized degree**.

Supplementary Table 3 **Mean ± SD values based on IQ, for the normalized betweenness centrality**.

Supplementary Table 4 **Mean ± SD values based on IQ, for the normalized eccentricity**.

Supplementary Table 5 **Statistical analysis results based on IQ for the main network metrics**.

Supplementary Table 6 **Statistical analysis results based on males IQ for the main network metrics**.

Supplementary Table 7 **Statistical analysis results between high-IQ males and females for the main network metrics**.
