# Corrigendum: Resting-State Functional Connectivity and Network Analysis of Cerebellum With Respect to IQ and Gender

**DOI:** 10.3389/fnhum.2018.00216

**Published:** 2018-05-28

**Authors:** Vasileios C. Pezoulas, Michalis Zervakis, Sifis Michelogiannis, Manousos A. Klados

**Affiliations:** ^1^School of Electrical and Computer Engineering, Technical University of Crete, Chania, Greece; ^2^Neurophysiological Research Laboratory (L. Widén), School of Medicine, University of Crete, Heraklion, Greece; ^3^Max Planck Research Group for Neuroanatomy and Connectivity, Max Planck Institute for Human Cognitive and Brain Sciences, Leipzig, Germany

**Keywords:** cerebellum, fMRI, small-world network, minimum spanning tree, IQ, median response time

In the original article, there was an error. We used the crystallized IQ term instead of the term fluid intelligence. This mistake does not affect the overall interpretation of our results since both of these terms are the same as far as the education is concerned. We have simply replaced the “crystallized” term with the “fluid” term within the original article.

A correction has been made to the title, which was originally Resting-State Functional Connectivity and Network Analysis of Cerebellum With Respect to Crystallized IQ and Gender. It has been updated to the following:

Resting-State Functional Connectivity and Network Analysis of Cerebellum with Respect to IQ and Gender.

A correction has been made to the abstract which has been updated to the following:

During the last years, it has been established that the prefrontal and posterior parietal brain lobes, which are mostly related to intelligence, have many connections to cerebellum. However, there is a limited research investigating cerebellum's relationship with cognitive processes. In this study, the network of cerebellum was analyzed in order to investigate its overall organization in individuals with low and high fluid Intelligence Quotient (IQ). Functional magnetic resonance imaging (fMRI) data were selected from 136 subjects in resting-state from the Human Connectome Project (HCP) database and were further separated into two IQ groups composed of 69 low-IQ and 67 high-IQ subjects. Cerebellum was parcellated into 28 lobules/ROIs (per subject) using a standard cerebellum anatomical atlas. Thereafter, correlation matrices were constructed by computing Pearson's correlation coefficients between the average BOLD time-series for each pair of ROIs inside the cerebellum. By computing conventional graph metrics, small-world network properties were verified using the weighted clustering coefficient and the characteristic path length for estimating the trade-off between segregation and integration. In addition, a connectivity metric was computed for extracting the average cost per network. The concept of the Minimum Spanning Tree (MST) was adopted and implemented in order to avoid methodological biases in graph comparisons and retain only the strongest connections per network. Subsequently, six global and three local metrics were calculated in order to retrieve useful features concerning the characteristics of each MST. Moreover, the local metrics of degree and betweenness centrality were used to detect hubs, i.e., nodes with high importance. The computed set of metrics gave rise to extensive statistical analysis in order to examine differences between low and high-IQ groups, as well as between all possible gender-based group combinations. Our results reveal that both male and female networks have small-world properties with differences in females (especially in higher IQ females) indicative of higher neural efficiency in cerebellum. There is a trend toward the same direction in men, but without significant differences. Finally, three lobules showed maximum correlation with the median response time in low-IQ individuals, implying that there is an increased effort dedicated locally by this population in cognitive tasks.

A correction has been made to the Materials and Methods section, subsection Subjects:

Our data was collected from the Human Connectome Project (HCP) database, an open-source database aiming to provide deep examination of the human brain connectome (Van Essen et al., 2013). The HCP is the result of efforts of co-investigators from the University of California, Los Angeles, Martinos Center for Biomedical Imaging at Massachusetts General Hospital (MGH), Washington University, and the University of Minnesota. The present study analyzes rs-fMRI data collected from the HCP database after the HCP S500 + MEG2 data release, between the first six quarterly releases (Q1–Q6), with few cases also collected in Q7 and later. Functional magnetic resonance imaging (fMRI) data was initially acquired from 492 healthy subjects at rest with eyes open with relaxed fixation on a projected bright cross-hair on a dark background (and presented in a darkened room) (Van Essen et al., 2013). All subjects with psychiatric history, extensive substance use and hard alcohol history have been removed since the cerebellum is heavily impacted by alcohol abuse/dependence (Sullivan et al., 2010) and there is also evidence to suggest that the cerebellum is impacted by marijuana as well (Block et al., 2000; Lopez-Larson et al., 2011; Solowij et al., 2011). Moreover, additional information related to siblings and twins have been obtained. The population has been restricted to only one member of a sibling/twin pair so as to overcome shared variance issues. Fluid IQ scores were obtained per subject prior to scanning. Finally, subjects were separated based on their fluid IQ scores into two groups as described in the following section.

A correction has been made to the Materials and Methods section, subsection Groups Formation:

Crystallized intelligence is conceptualized as the product of experience, both cultural and educational, in interaction with fluid intelligence, which implies the existence of an intersection between crystallized and fluid intelligence as far as the educational experience is (exclusively) concerned (Barch et al., 2013; Happé, 2013; Schipolowski et al., 2014). The HCP database provides fluid intelligence measures obtained using a Form-A of an abbreviated version of the Raven's patterns, developed by Gur and colleagues (Bilker et al., 2012; Barch et al., 2013). More specifically, participants were presented with patterns made up of 2 × 2, 3 × 3, or 5 × 5 arrangements of squares, with one of the squares missing. Each participant must pick one of five response choices that best fits the missing square on the pattern. The task has 24 items and 3 bonus items, arranged in order of increasing difficulty. However, the task discontinues if the participant makes 5 incorrect responses in a row. Median response times (MRTs) were also collected per subject in order to study associations with brain measures.

The Keywords have also been updated to reflect this change.

The authors apologize for this error and state that this does not change the scientific conclusions of the article in any way.

The original article has been updated.

## Conflict of interest statement

The authors declare that the research was conducted in the absence of any commercial or financial relationships that could be construed as a potential conflict of interest.
