# Supplementary figures and images for: Resting-State Functional Connectivity and Network Analysis of Cerebellum with Respect to IQ and Gender
Source: Front Hum Neurosci. 2017 Apr 26;11:189. doi: 10.3389/fnhum.2017.00189 (PMC5405083; doi:10.3389/fnhum.2017.00189)

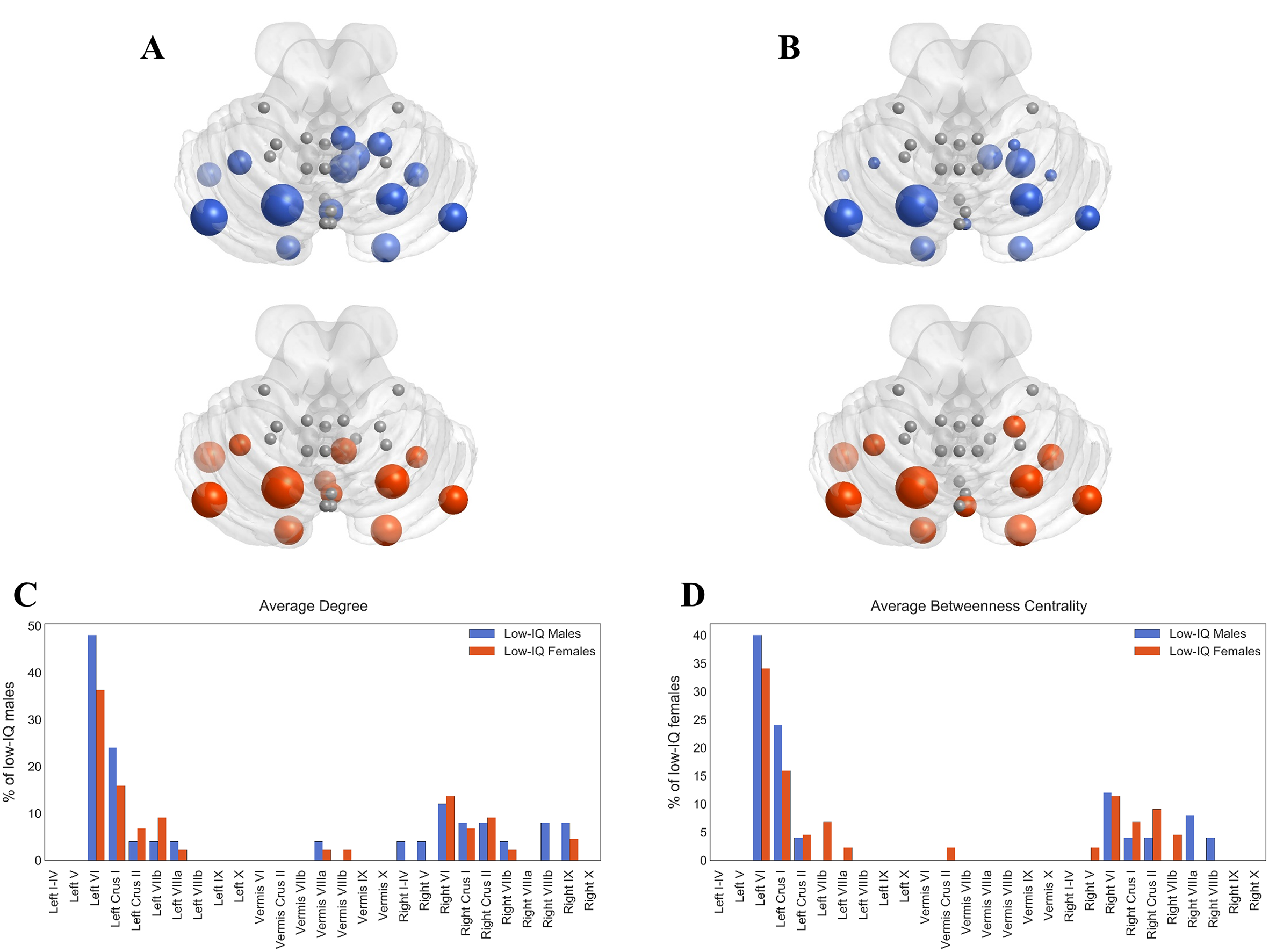

Supplement: Supplementary Figure 1 — Hub locations on cerebellum for low-IQ males (light blue) and females (orange) based on BC (A) and DEG (B). The size of each node depends on the percentage of low-IQ males and females with the highest BC (C) and DEG (D) values, respectively. [file Image1.TIF]

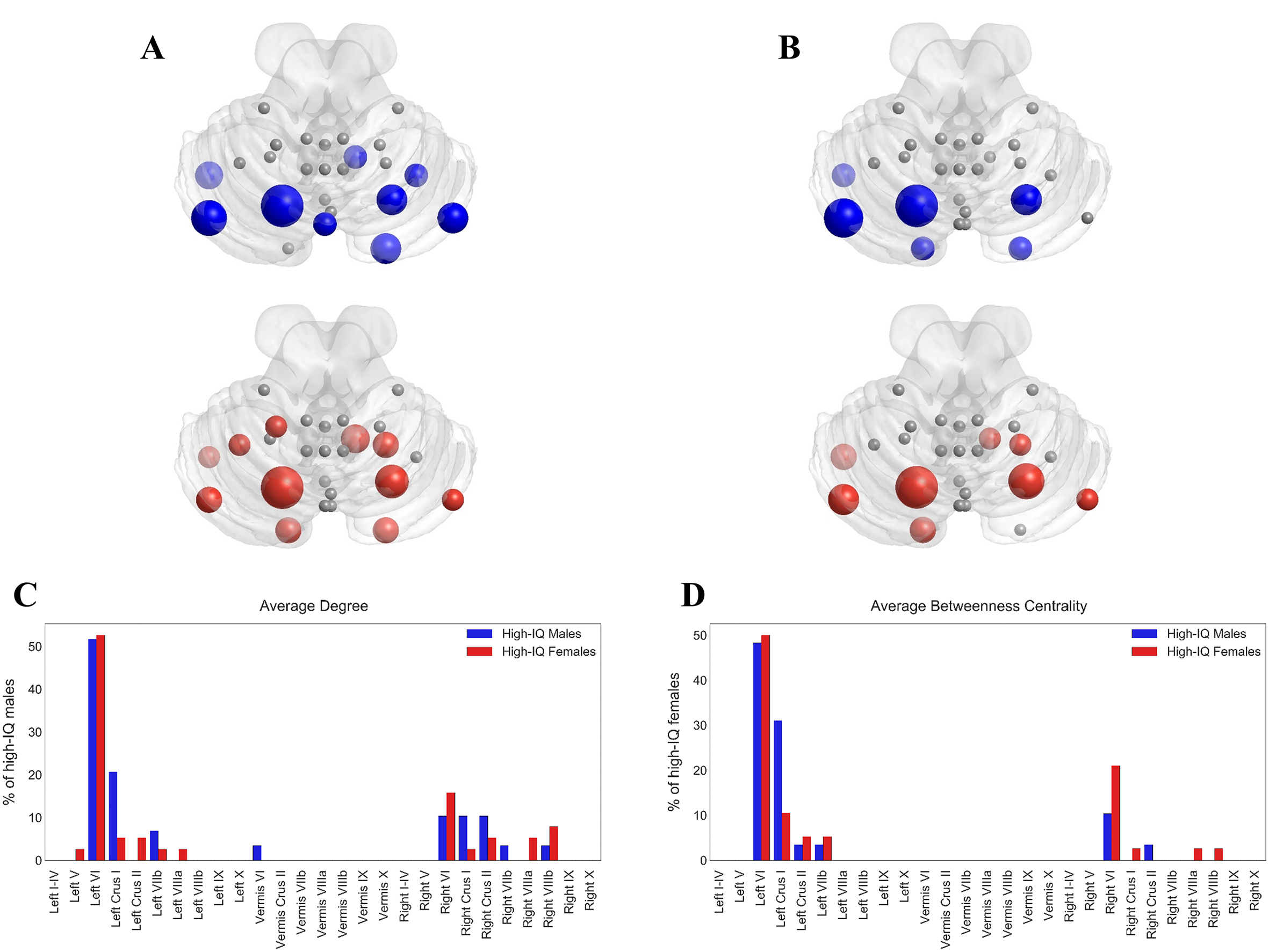

Supplement: Supplementary Figure 2 — Hub locations on cerebellum for high-IQ males (blue) and females (red) based on BC (A) and DEG (B). The size of each node depends on the percentage of high-IQ males and females with the highest BC (C) and DEG (D) values, respectively. [file Image2.TIF]

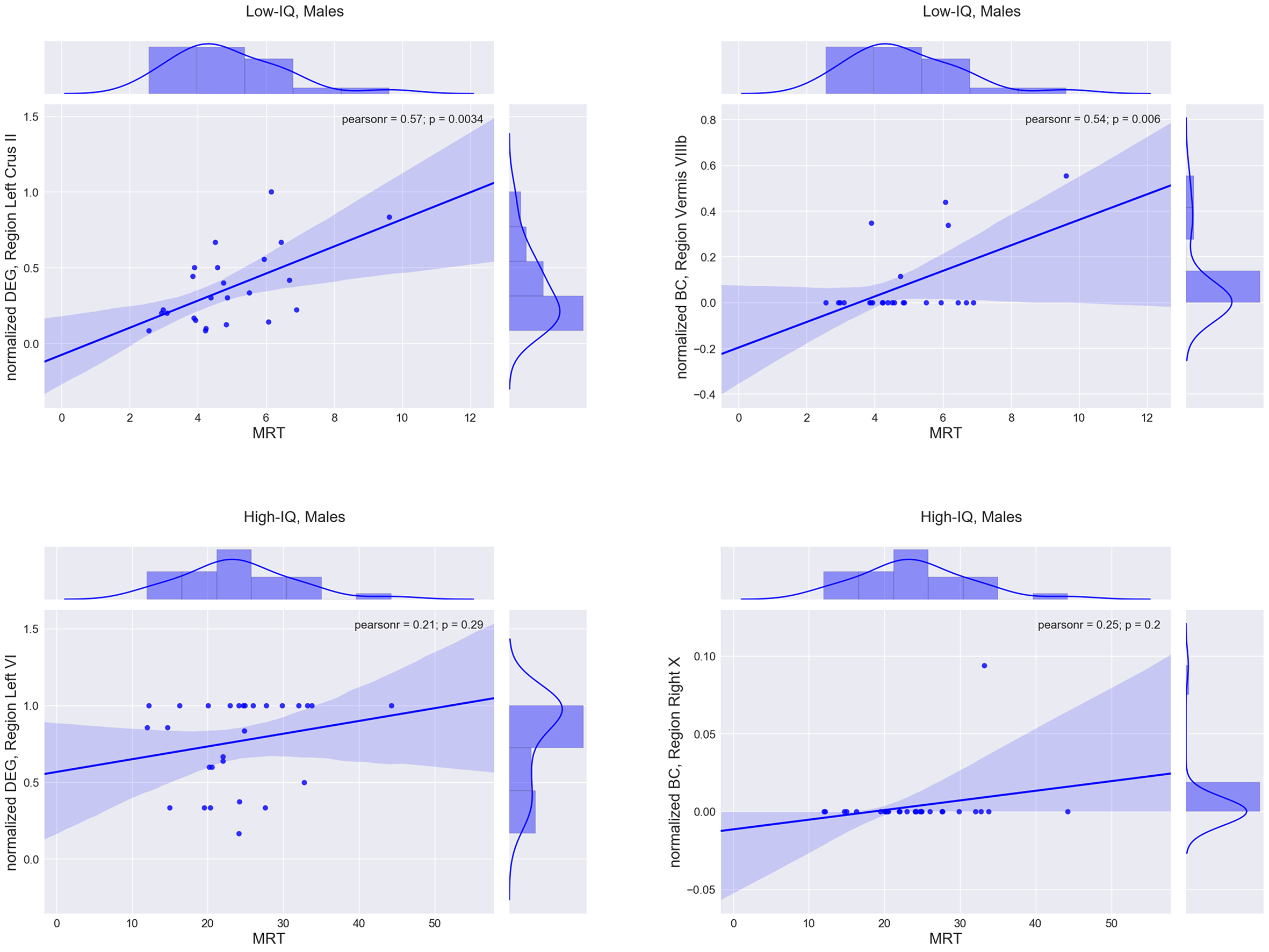

Supplement: Supplementary Figure 3 — Regions with the maximum correlation between average DEG or BC measure and median response times (MRTs) for low and high-IQ males. [file Image3.TIF]

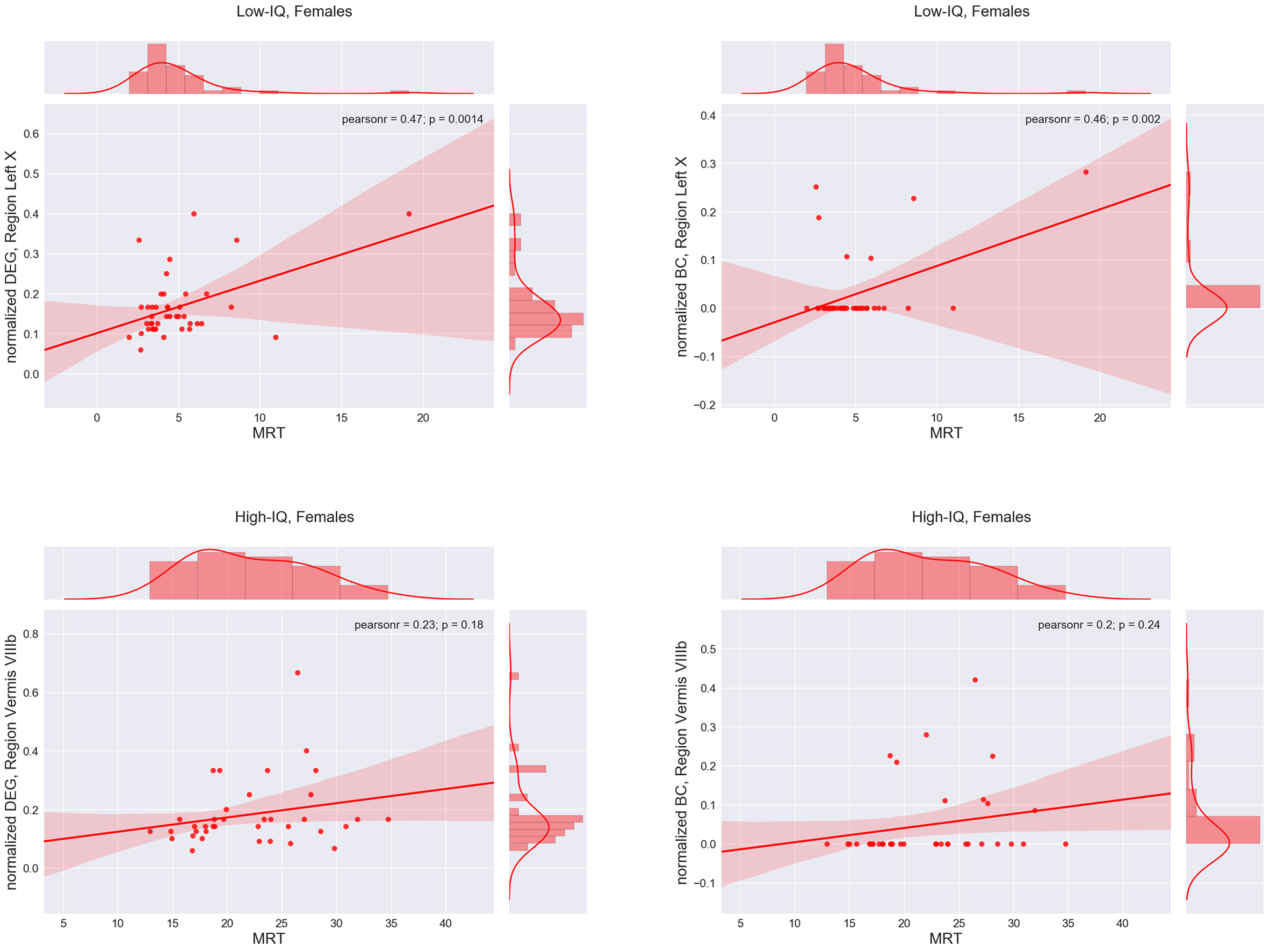

Supplement: Supplementary Figure 4 — Regions with the maximum correlation between average DEG or BC measure and median response times (MRTs) for low and high-IQ females. [file Image4.TIF]
