# Supplementary material for: Resting-State Functional Connectivity and Network Analysis of Cerebellum with Respect to IQ and Gender
Source: Front Hum Neurosci. 2017 Apr 26;11:189. doi: 10.3389/fnhum.2017.00189 (PMC5405083; doi:10.3389/fnhum.2017.00189)
Supplement: Supplementary Table 1 — Small-world properties information. [file Table1.DOCX]

| Supplementary Table 1. Small-world properties information. | | | |
| --- | --- | --- | --- |
| Groups | **Avg. clustering coefficient**  Mean±SD | **Characteristic path length**  Mean±SD | **Small-worldness**  Mean±SD |
| Low-IQ | 1.1939±0.0857 | 0.9548±0.0917 | 1.2644±0.1765 |
| High-IQ | 1.1634±0.0564 | 0.9640±0.0689 | 1.2126±0.1010 |
| Males, low-IQ | 1.1671±0.0661 | 0.9523±0.0774 | 1.2334±0.1243 |
| Males, high-IQ | 1.1520±0.0396 | 0.9454±0.0878 | 1.2287±0.1243 |
| Females, low-IQ | 1.2092±0.0923 | 0.9562±0.0997 | 1.2821±0.1994 |
| Females, high-IQ | 1.1720±0.0657 | 0.9781±0.0466 | 1.2002±0.0783 |
