# Supplementary material for: Resting-State Functional Connectivity and Network Analysis of Cerebellum with Respect to IQ and Gender
Source: Front Hum Neurosci. 2017 Apr 26;11:189. doi: 10.3389/fnhum.2017.00189 (PMC5405083; doi:10.3389/fnhum.2017.00189)
Supplement: Supplementary Table 2 — Mean ± SD values based on IQ, for the normalized degree. [file Table2.DOCX]

| Supplementary Table 2. Mean±SD values based on IQ, for the normalized degree. | | | | | | | |
| --- | --- | --- | --- | --- | --- | --- | --- |
| ROI | **Location**  **(Name)** | **Low-IQ**  Mean±SD | **High-IQ**  Mean±SD | **Low-IQ**  **Males**  Mean±SD | **High-IQ**  **Males**  Mean±SD | **Low-IQ**  **Females**  Mean±SD | **High-IQ**  **Females**  Mean±SD |
| 1 | Left I-IV | 0.1837±0.1029 | 0.1813±0.0967 | 0.1851±0.1005 | 0.1663±0.0774 | 0.1829±0.1054 | 0.1927±0.1088 |
| 2 | Left V | 0.1951±0.1002 | 0.1704±0.1256 | 0.2147±0.1274 | 0.1505±0.0556 | 0.1839±0.0805 | 0.1856±0.1589 |
| 3 | Left VI | 0.6859±0.3066 | 0.7566±0.3007 | 0.6738±0.3511 | 0.7733±0.2837 | 0.6928±0.2823 | 0.7439±0.3162 |
| 4 | Left Crus I | 0.5616±0.2928 | 0.5477±0.2716 | 0.5497±0.3324 | 0.5838±0.2948 | 0.5683±0.2715 | 0.5202±0.2530 |
| 5 | Left Crus II | 0.3543±0.2488 | 0.3487±0.2391 | 0.3580±0.2425 | 0.3219±0.2225 | 0.3522±0.2550 | 0.3691±0.2520 |
| 6 | Left VIIb | 0.3652±0.2874 | 0.3183±0.2355 | 0.2835±0.2250 | 0.3188±0.2417 | 0.4116±0.3103 | 0.3179±0.2338 |
| 7 | Left VIIIa | 0.3176±0.2200 | 0.2837±0.1853 | 0.3228±0.2420 | 0.2320±0.1253 | 0.3147±0.2094 | 0.3232±0.2138 |
| 8 | Left VIIIb | 0.1718±0.0895 | 0.1472±0.0762 | 0.1552±0.0637 | 0.1353±0.0452 | 0.1813±0.1007 | 0.1562±0.0929 |
| 9 | Left IX | 0.1791±0.1043 | 0.1897±0.1291 | 0.1930±0.1373 | 0.1898±0.1248 | 0.1712±0.0808 | 0.1896±0.1340 |
| 10 | Left X | 0.1579±0.0715 | 0.1456±0.0590 | 0.1427±0.0544 | 0.1528±0.0635 | 0.1665±0.0789 | 0.1402±0.0555 |
| 11 | Vermis VI | 0.2037±0.1334 | 0.1763±0.1427 | 0.1819±0.1376 | 0.1976±0.1936 | 0.2161±0.1309 | 0.1601±0.0858 |
| 12 | Vermis Crus II | 0.2056±0.1251 | 0.1832±0.0958 | 0.1827±0.0876 | 0.1896±0.0965 | 0.2186±0.1414 | 0.1784±0.0963 |
| 13 | Vermis VIIb | 0.1481±0.0639 | 0.1378±0.0496 | 0.1539±0.0911 | 0.1373±0.0599 | 0.1447±0.0424 | 0.1382±0.0408 |
| 14 | Vermis VIIIa | 0.2550±0.2053 | 0.1972±0.1200 | 0.2493±0.2217 | 0.1923±0.1267 | 0.2582±0.1979 | 0.2009±0.1163 |
| 15 | Vermis VIIIb | 0.2215±0.1864 | 0.1730±0.1019 | 0.2117±0.1948 | 0.1642±0.0808 | 0.2271±0.1835 | 0.1797±0.1161 |
| 16 | Vermis IX | 0.2406±0.1565 | 0.2188±0.1484 | 0.2208±0.1516 | 0.2172±0.1661 | 0.2519±0.1598 | 0.2201±0.1357 |
| 17 | Vermis X | 0.1551±0.0755 | 0.1367±0.0407 | 0.1522±0.0869 | 0.1347±0.0412 | 0.1567±0.0692 | 0.1382±0.0408 |
| 18 | Right I-IV | 0.2176±0.1693 | 0.1731±0.0767 | 0.2467±0.2286 | 0.1761±0.0780 | 0.2011±0.1240 | 0.1708±0.0767 |
| 19 | Right V | 0.2046±0.1500 | 0.1664±0.0941 | 0.1987±0.1869 | 0.1520±0.0628 | 0.2079±0.1267 | 0.1774±0.1119 |
| 20 | Right VI | 0.4341±0.3026 | 0.4681±0.2960 | 0.3848±0.3069 | 0.4095±0.2826 | 0.4621±0.3001 | 0.5128±0.3018 |
| 21 | Right Crus I | 0.4310±0.2554 | 0.3970±0.2338 | 0.3644±0.2501 | 0.4780±0.2655 | 0.4688±0.2534 | 0.3352±0.1875 |
| 22 | Right Crus II | 0.3338±0.2621 | 0.3227±0.2529 | 0.2929±0.2571 | 0.3371±0.2813 | 0.3570±0.2650 | 0.3116±0.2322 |
| 23 | Right VIIb | 0.2567±0.1945 | 0.2269±0.1729 | 0.2572±0.2067 | 0.2384±0.2195 | 0.2565±0.1897 | 0.2180±0.1292 |
| 24 | Right VIIIa | 0.2775±0.2046 | 0.2833±0.2037 | 0.3061±0.2083 | 0.2250±0.1420 | 0.2612±0.2031 | 0.3278±0.2325 |
| 25 | Right VIIIb | 0.1921±0.1728 | 0.2109±0.2203 | 0.2538±0.2621 | 0.1727±0.1679 | 0.1570±0.0734 | 0.2400±0.2514 |
| 26 | Right IX | 0.3009±0.2348 | 0.2095±0.1398 | 0.3147±0.2445 | 0.1995±0.1515 | 0.2931±0.2316 | 0.2170±0.1317 |
| 27 | Right X | 0.1532±0.0727 | 0.1436±0.0479 | 0.1382±0.0522 | 0.1342±0.0385 | 0.1618±0.0814 | 0.1507±0.0534 |
