# Supplementary material for: Resting-State Functional Connectivity and Network Analysis of Cerebellum with Respect to IQ and Gender
Source: Front Hum Neurosci. 2017 Apr 26;11:189. doi: 10.3389/fnhum.2017.00189 (PMC5405083; doi:10.3389/fnhum.2017.00189)
Supplement: Supplementary Table 3 — Mean ± SD values based on IQ, for the normalized betweenness centrality. [file Table3.DOCX]

| Supplementary Table 3. Mean±SD values based on IQ, for the normalized betweenness centrality. | | | | | | | |
| --- | --- | --- | --- | --- | --- | --- | --- |
| ROI | **Location**  **(Name)** | **Low-IQ**  Mean±SD | **High-IQ**  Mean±SD | **Low-IQ**  **Males**  Mean±SD | **High-IQ**  **Males**  Mean±SD | **Low-IQ**  **Females**  Mean±SD | **High-IQ**  **Females**  Mean±SD |
| 1 | Left I-IV | 0.0354±0.0758 | 0.0391±0.0640 | 0.0382±0.0610 | 0.0322±0.0554 | 0.0338±0.0837 | 0.0444±0.0702 |
| 2 | Left V | 0.0523±0.0823 | 0.0401±0.1071 | 0.0736±0.0910 | 0.0230±0.0593 | 0.0402±0.0753 | 0.0530±0.1320 |
| 3 | Left VI | 0.6693±0.3542 | 0.7198±0.3628 | 0.6835±0.3673 | 0.7387±0.3587 | 0.6613±0.3506 | 0.7054±0.3700 |
| 4 | Left Crus I | 0.6002±0.3346 | 0.5808±0.3230 | 0.5613±0.3855 | 0.6504±0.3275 | 0.6223±0.3045 | 0.5278±0.3135 |
| 5 | Left Crus II | 0.3147±0.3198 | 0.3242±0.3063 | 0.3045±0.3013 | 0.3081±0.3276 | 0.3205±0.3331 | 0.3364±0.2928 |
| 6 | Left VIIb | 0.2989±0.3353 | 0.2775±0.3358 | 0.2105±0.2753 | 0.2482±0.3078 | 0.3492±0.3582 | 0.3000±0.3582 |
| 7 | Left VIIIa | 0.2084±0.2553 | 0.1722±0.2192 | 0.2226±0.2594 | 0.1060±0.1527 | 0.2003±0.2556 | 0.2228±0.2490 |
| 8 | Left VIIIb | 0.0403±0.1259 | 0.0143±0.0714 | 0.0214±0.0714 | 0.0039±0.0208 | 0.0511±0.1480 | 0.0223±0.0928 |
| 9 | Left IX | 0.0413±0.0893 | 0.0647±0.1459 | 0.0695±0.1228 | 0.0619±0.1528 | 0.0253±0.0589 | 0.0669±0.1425 |
| 10 | Left X | 0.0184±0.0594 | 0.0180±0.0712 | 0.0044±0.0218 | 0.0375±0.1038 | 0.0263±0.0717 | 0.0031±0.0189 |
| 11 | Vermis VI | 0.0784±0.1598 | 0.0529±0.1699 | 0.0727±0.1858 | 0.0774±0.2120 | 0.0816±0.1453 | 0.0342±0.1290 |
| 12 | Vermis Crus II | 0.0569±0.1318 | 0.0383±0.0571 | 0.0358±0.0557 | 0.0462±0.0609 | 0.0689±0.1592 | 0.0322±0.0541 |
| 13 | Vermis VIIb | 0.0033±0.0191 | 0.0031±0.0176 | 0.0090±0.0313 | 0.0036±0.0193 | 0±0 | 0.0027±0.0164 |
| 14 | Vermis VIIIa | 0.1016±0.1695 | 0.0620±0.1033 | 0.0974±0.1742 | 0.0731±0.1238 | 0.1039±0.1687 | 0.0535±0.0852 |
| 15 | Vermis VIIIb | 0.0744±0.1537 | 0.0412±0.0851 | 0.0716±0.1604 | 0.0339±0.0638 | 0.0759±0.1516 | 0.0467±0.0988 |
| 16 | Vermis IX | 0.0891±0.1293 | 0.0723±0.1088 | 0.0919±0.1616 | 0.0689±0.1118 | 0.0875±0.1088 | 0.0748±0.1079 |
| 17 | Vermis X | 0.0167±0.0750 | 0.0032±0.0185 | 0.0251±0.1065 | 0.0040±0.0213 | 0.0119±0.0500 | 0.0027±0.0164 |
| 18 | Right I-IV | 0.0626±0.1178 | 0.0404±0.0747 | 0.0883±0.1634 | 0.0381±0.0612 | 0.0479±0.0801 | 0.0421±0.0844 |
| 19 | Right V | 0.0652±0.1619 | 0.0355±0.0877 | 0.0637±0.1718 | 0.0199±0.0460 | 0.0661±0.1580 | 0.0475±0.1086 |
| 20 | Right VI | 0.3834±0.3767 | 0.3863±0.3631 | 0.3321±0.3785 | 0.3111±0.3262 | 0.4126±0.3768 | 0.4437±0.3832 |
| 21 | Right Crus I | 0.3979±0.3166 | 0.3820±0.2797 | 0.3026±0.2970 | 0.4589±0.2971 | 0.4521±0.3178 | 0.3234±0.2542 |
| 22 | Right Crus II | 0.2789±0.3339 | 0.2405±0.2795 | 0.1991±0.2776 | 0.2228±0.2722 | 0.3242±0.3571 | 0.2540±0.2878 |
| 23 | Right VIIb | 0.1880±0.2807 | 0.1696±0.2706 | 0.1811±0.2332 | 0.1492±0.2568 | 0.1920±0.3069 | 0.1851±0.2831 |
| 24 | Right VIIIa | 0.1618±0.2700 | 0.1920±0.2653 | 0.2106±0.2965 | 0.0952±0.1775 | 0.1340±0.2530 | 0.2658±0.2980 |
| 25 | Right VIIIb | 0.0509±0.1721 | 0.0725±0.2041 | 0.1211±0.2647 | 0.0306±0.1091 | 0.0110±0.0569 | 0.1044±0.2507 |
| 26 | Right IX | 0.1540±0.2148 | 0.0726±0.1333 | 0.1984±0.2233 | 0.0707±0.1525 | 0.1288±0.2082 | 0.0741±0.1187 |
| 27 | Right X | 0.0071±0.0287 | 0.0128±0.0447 | 0±0 | 0.0032±0.0175 | 0.0111±0.0355 | 0.0200±0.0567 |
