# Supplementary material for: Resting-State Functional Connectivity and Network Analysis of Cerebellum with Respect to IQ and Gender
Source: Front Hum Neurosci. 2017 Apr 26;11:189. doi: 10.3389/fnhum.2017.00189 (PMC5405083; doi:10.3389/fnhum.2017.00189)
Supplement: Supplementary Table 4 — Mean ± SD values based on IQ, for the normalized eccentricity. [file Table4.DOCX]

| Supplementary Table 4. Mean±SD values based on IQ, for the normalized eccentricity. | | | | | | | |
| --- | --- | --- | --- | --- | --- | --- | --- |
| ROI | **Location**  **(Name)** | **Low-IQ**  Mean±SD | **High-IQ**  Mean±SD | **Low-IQ**  **Males**  Mean±SD | **High-IQ**  **Males**  Mean±SD | **Low-IQ**  **Females**  Mean±SD | **High-IQ**  **Females**  Mean±SD |
| 1 | Left I-IV | 0.8062±0.1066 | 0.7970±0.0971 | 0.8062±0.1066 | 0.7970±0.0971 | 0.8128±0.1090 | 0.7889±0.0928 |
| 2 | Left V | 0.7509±0.1173 | 0.7650±0.1225 | 0.7509±0.1173 | 0.7650±0.1225 | 0.7545±0.1164 | 0.7493±0.1204 |
| 3 | Left VI | 0.6315±0.1008 | 0.6349±0.1039 | 0.6315±0.1008 | 0.6349±0.1039 | 0.6312±0.0996 | 0.6340±0.1162 |
| 4 | Left Crus I | 0.6444±0.1191 | 0.6586±0.1023 | 0.6444±0.1191 | 0.6586±0.1023 | 0.6324±0.1129 | 0.6802±0.1108 |
| 5 | Left Crus II | 0.6668±0.1134 | 0.6777±0.0984 | 0.6668±0.1134 | 0.6777±0.0984 | 0.6516±0.1020 | 0.6912±0.1079 |
| 6 | Left VIIb | 0.6873±0.0893 | 0.6860±0.0999 | 0.6873±0.0893 | 0.6860±0.0999 | 0.6714±0.0948 | 0.6765±0.1091 |
| 7 | Left VIIIa | 0.7198±0.0967 | 0.7135±0.0967 | 0.7198±0.0967 | 0.7135±0.0967 | 0.7158±0.0977 | 0.6885±0.0992 |
| 8 | Left VIIIb | 0.7863±0.1085 | 0.7872±0.1185 | 0.7863±0.1085 | 0.7872±0.1185 | 0.7906±0.1093 | 0.7620±0.1183 |
| 9 | Left IX | 0.7657±0.1169 | 0.7721±0.1259 | 0.7657±0.1169 | 0.7721±0.1259 | 0.7619±0.1126 | 0.7981±0.1340 |
| 10 | Left X | 0.7958±0.1280 | 0.7869±0.1225 | 0.7958±0.1280 | 0.7869±0.1225 | 0.7875±0.1340 | 0.7926±0.1083 |
| 11 | Vermis VI | 0.7245±0.1106 | 0.7577±0.1146 | 0.7245±0.1106 | 0.7577±0.1146 | 0.7192±0.1092 | 0.7592±0.1171 |
| 12 | Vermis Crus II | 0.7952±0.1220 | 0.7844±0.1117 | 0.7952±0.1220 | 0.7844±0.1117 | 0.7967±0.1126 | 0.7773±0.1199 |
| 13 | Vermis VIIb | 0.9276±0.0881 | 0.9327±0.0841 | 0.9276±0.0881 | 0.9327±0.0841 | 0.9294±0.0970 | 0.9188±0.0946 |
| 14 | Vermis VIIIa | 0.7640±0.1184 | 0.7847±0.1217 | 0.7640±0.1184 | 0.7847±0.1217 | 0.7716±0.1258 | 0.7873±0.1088 |
| 15 | Vermis VIIIb | 0.8405±0.1239 | 0.8392±0.1157 | 0.8405±0.1239 | 0.8392±0.1157 | 0.8414±0.1233 | 0.8384±0.1255 |
| 16 | Vermis IX | 0.8039±0.1264 | 0.8116±0.1156 | 0.8039±0.1264 | 0.8116±0.1156 | 0.8036±0.1214 | 0.8031±0.1182 |
| 17 | Vermis X | 0.8956±0.1198 | 0.9371±0.0843 | 0.8956±0.1198 | 0.9371±0.0843 | 0.9064±0.1096 | 0.9356±0.0816 |
| 18 | Right I-IV | 0.8099±0.1129 | 0.8003±0.1182 | 0.8099±0.1129 | 0.8003±0.1182 | 0.8114±0.1050 | 0.8078±0.1300 |
| 19 | Right V | 0.7646±0.1153 | 0.7665±0.1174 | 0.7646±0.1153 | 0.7665±0.1174 | 0.7645±0.1140 | 0.7555±0.1241 |
| 20 | Right VI | 0.6665±0.1017 | 0.6677±0.1006 | 0.6665±0.1017 | 0.6677±0.1006 | 0.6690±0.1019 | 0.6674±0.1109 |
| 21 | Right Crus I | 0.6663±0.1005 | 0.6803±0.1029 | 0.6663±0.1005 | 0.6803±0.1029 | 0.6593±0.1054 | 0.7071±0.1028 |
| 22 | Right Crus II | 0.6818±0.1049 | 0.7081±0.0980 | 0.6818±0.1049 | 0.7081±0.0980 | 0.6782±0.1104 | 0.7292±0.0988 |
| 23 | Right VIIb | 0.7056±0.0951 | 0.7112±0.1105 | 0.7056±0.0951 | 0.7112±0.1105 | 0.7043±0.0991 | 0.7051±0.1136 |
| 24 | Right VIIIa | 0.7266±0.1035 | 0.7140±0.1019 | 0.7266±0.1035 | 0.7140±0.1019 | 0.7189±0.0769 | 0.6947±0.1105 |
| 25 | Right VIIIb | 0.7770±0.1202 | 0.7763±0.0987 | 0.7770±0.1202 | 0.7763±0.0987 | 0.7642±0.1097 | 0.7839±0.1076 |
| 26 | Right IX | 0.7378±0.1057 | 0.7739±0.1030 | 0.7378±0.1057 | 0.7739±0.1030 | 0.7487±0.1121 | 0.7880±0.0936 |
| 27 | Right X | 0.8061±0.1106 | 0.7831±0.0996 | 0.8061±0.1106 | 0.7831±0.0996 | 0.7945±0.1081 | 0.7713±0.1066 |
