# Supplementary material for: Resting-State Functional Connectivity and Network Analysis of Cerebellum with Respect to IQ and Gender
Source: Front Hum Neurosci. 2017 Apr 26;11:189. doi: 10.3389/fnhum.2017.00189 (PMC5405083; doi:10.3389/fnhum.2017.00189)
Supplement: Supplementary Table 5 — Statistical analysis results based on IQ for the main network metrics. [file Table5.DOCX]

| Supplementary Table 5. Statistical analysis results based on IQ for the main network metrics. | | | | |
| --- | --- | --- | --- | --- |
| Metric | **Low-IQ**  Mean±SD | **High-IQ**  Mean±SD | **F** | **p** |
| $\boldsymbol{C}_{\boldsymbol{w}}$ | 1.1939±0.0857 | 1.1634±0.0564 | 5.8769 | **0.0167** |
| $\boldsymbol{L}_{\boldsymbol{w}}$ | 0.9548±0.0917 | 0.9640±0.0689 | 0.6234 | 0.4312 |
| $\boldsymbol{\sigma}^{\boldsymbol{w}}$ | 1.2644±0.1765 | 1.2126±0.1010 | 3.6449 | 0.0584 |
| $\boldsymbol{conn}$ | 0.1784±0.0763 | 0.2073±0.0878 | 5.1324 | **0.0251** |
| $\boldsymbol{d}$ | 0.4002±0.1632 | 0.3376±0.1215 | 5.2927 | **0.0230** |
| $\boldsymbol{r}$ | 0.4101±0.1641 | 0.3540±0.1400 | 4.3788 | **0.0383** |
| $\boldsymbol{L}_{\boldsymbol{f}}$ | 0.5920±0.0943 | 0.6171±0.0872 | 2.7130 | 0.1019 |
| $\boldsymbol{T}_{\boldsymbol{h}}$ | 0.2960±0.0471 | 0.3086±0.0436 | 2.7130 | 0.1019 |
| $\boldsymbol{\kappa}$ | 2.2800±0.3344 | 2.3192±0.2833 | 0.7636 | 0.3838 |
| $\boldsymbol{r}_{\boldsymbol{deg}}$ | -0.3438±0.1262 | -0.3742±0.1233 | 2.5276 | 0.1142 |
| with bold highlight: statistical significant results $\boldsymbol{(p<0.05)}$. | | | | |
