# Supplementary material for: Resting-State Functional Connectivity and Network Analysis of Cerebellum with Respect to IQ and Gender
Source: Front Hum Neurosci. 2017 Apr 26;11:189. doi: 10.3389/fnhum.2017.00189 (PMC5405083; doi:10.3389/fnhum.2017.00189)
Supplement: Supplementary Table 6 — Statistical analysis results based on males IQ for the main network metrics. [file Table6.DOCX]

| Supplementary Table 6. Statistical analysis results based on males IQ for the main network metrics. | | | | |
| --- | --- | --- | --- | --- |
| Metric | **Low-IQ**  **Males**  Mean±SD | **High-IQ**  **Males**  Mean±SD | **F** | **p** |
| $\boldsymbol{C}_{\boldsymbol{w}}$ | 1.1671±0.0661 | 1.1520±0.0396 | 0.9639 | 0.3308 |
| $\boldsymbol{L}_{\boldsymbol{w}}$ | 0.9523±0.0774 | 0.9454±0.0878 | 0.1237 | 0.7265 |
| $\boldsymbol{\sigma}^{\boldsymbol{w}}$ | 1.2334±0.1243 | 1.2287±0.1243 | 0.0191 | 0.8907 |
| $\boldsymbol{conn}$ | 0.2058±0.0822 | 0.2151±0.0916 | 0.2060 | 0.6518 |
| $\boldsymbol{d}$ | 0.5227±0.2231 | 0.4906±0.1740 | 0.0973 | 0.7563 |
| $\boldsymbol{r}$ | 0.5403±0.2298 | 0.5161±0.1895 | 0.0452 | 0.8325 |
| $\boldsymbol{L}_{\boldsymbol{f}}$ | 0.5892±0.0775 | 0.6313±0.0766 | 4.0361 | 0.0515 |
| $\boldsymbol{T}_{\boldsymbol{h}}$ | 0.2946±0.0387 | 0.3156±0.0383 | 4.0361 | 0.0515 |
| $\boldsymbol{\kappa}$ | 2.3391±0.4098 | 2.3229±0.3085 | 0.0001 | 0.9912 |
| $\boldsymbol{r}_{\boldsymbol{deg}}$ | -0.3374±0.1138 | -0.3709±0.1213 | 0.8741 | 0.3541 |
