# Supplementary material for: Resting-State Functional Connectivity and Network Analysis of Cerebellum with Respect to IQ and Gender
Source: Front Hum Neurosci. 2017 Apr 26;11:189. doi: 10.3389/fnhum.2017.00189 (PMC5405083; doi:10.3389/fnhum.2017.00189)
Supplement: Supplementary Table 7 — Statistical analysis results between high-IQ males and females for the main network metrics. [file Table7.DOCX]

| Supplementary Table 7. Statistical analysis results between high-IQ males and females for the main network metrics. | | | | |
| --- | --- | --- | --- | --- |
| Metric | **High-IQ**  **Males**  Mean±SD | **High-IQ**  **Females**  Mean±SD | **F** | **p** |
| $\boldsymbol{C}_{\boldsymbol{w}}$ | 1.1520±0.0396 | 1.1720±0.0657 | 1.9689 | 0.1653 |
| $\boldsymbol{L}_{\boldsymbol{w}}$ | 0.9454±0.0878 | 0.9781±0.0466 | 4.5376 | **0.0369** |
| $\boldsymbol{\sigma}^{\boldsymbol{w}}$ | 1.2287±0.1243 | 1.2002±0.0783 | 1.0741 | 0.3039 |
| $\boldsymbol{conn}$ | 0.2151±0.0916 | 0.2014±0.0856 | 0.4643 | 0.4980 |
| $\boldsymbol{d}$ | 0.5494±0.1948 | 0.5781±0.2116 | 0.2142 | 0.6450 |
| $\boldsymbol{r}$ | 0.3937±0.1446 | 0.4174±0.1736 | 0.2158 | 0.6438 |
| $\boldsymbol{L}_{\boldsymbol{f}}$ | 0.6313±0.0766 | 0.6063±0.0940 | 1.6029 | 0.2100 |
| $\boldsymbol{T}_{\boldsymbol{h}}$ | 0.3156±0.0383 | 0.3031±0.0470 | 1.6029 | 0.2100 |
| $\boldsymbol{\kappa}$ | 2.3229±0.3085 | 2.3164±0.2666 | 0.0002 | 0.9877 |
| $\boldsymbol{r}_{\boldsymbol{deg}}$ | -0.3709±0.1213 | -0.3766±0.1264 | 0.0516 | 0.8210 |
| with bold highlight: statistical significant results $\boldsymbol{(p<0.05)}$. | | | | |
